# Plasticity of metabolism in plants

**DOI:** 10.1111/tpj.71159

**Published:** 2026-10-12

**Authors:** Xufeng Chen, Yinan Ouyang, Ashwin Ananthanarayanan, Dhanush S. Srikanthan, Chibueze K. Ene, Arsalan Khan, Joerg Fettke, Mustafa Bulut, Zoran Nikoloski, Sandra Marcela Correa, Alisdair R. Fernie

**Affiliations:** ^1^ Max Planck Institute of Molecular Plant Physiology Am Mühlenberg 1 14476 Golm, Potsdam Germany; ^2^ Bioinformatics Department Institute of Biochemistry and Biology, University of Potsdam Karl‐Liebknecht‐Str. 24‐25 14476 Potsdam Germany; ^3^ Biopolymer Analytics Department Institute of Biochemistry and Biology, University of Potsdam Karl‐Liebknecht‐Str. 24‐25 14476 Potsdam Germany; ^4^ Leibniz Institute of Plant Biochemistry (IPB) Weinberg 3 06120 Halle (Saale) Germany

**Keywords:** carbohydrate metabolism, lipid, plasticity, photorespiration, nitrogen fertilizer, reaction norm

## Abstract

The ability of plants to reprogram their metabolism in response to fluctuating environments, referred to as metabolic plasticity, is a key feature that enables sessile organisms to maintain growth, survival, and reproduction under changing conditions. As a mechanistic component of phenotypic plasticity, metabolic plasticity can buffer environmental perturbations and confer adaptive advantages. However, despite growing evidence for extensive metabolic remodeling under stress, the genetic and regulatory basis of this plasticity remains incompletely understood. Here, we review metabolic plasticity across major metabolic domains in plants, with emphasis on carbon, nitrogen, photorespiratory, and lipid metabolism, emphasizing how environmental cues and genetic regulation jointly shape metabolic responses. We conclude that, although metabolic plasticity is widespread in plants, its mechanistic and genetic basis remains incompletely understood; population‐scale studies and mathematical modeling offer promising avenues to resolve this gap and extend current insights from model systems to crops.

## Significance Statement

In this perspective, we provide a forward‐looking review on the study of the plasticity of plant metabolism paying particular attention to carbon, nitrogen, photorespiratory, and lipid metabolism. We attempt to describe strategies in which the relative effects of environmental and genetic plasticity can be quantified and how population‐scale genetics and mathematical modeling will likely play a prominent role in doing so.

## INTRODUCTION

Global climate change is increasing the frequency, intensity, and unpredictability of environmental fluctuations, including heat waves, drought episodes, and nutrient instability. For plants, which are sessile organisms, survival and reproductive success critically depend on the ability to adjust to these changing conditions *in situ*. A central mechanism enabling such adjustment is phenotypic plasticity, defined as the capacity of a single genotype to generate different phenotypes across environments. By translating genetic potential into context‐dependent phenotypic outcomes, plasticity provides a key functional link between genotype and environment. It is therefore a major determinant of plant resilience in heterogeneous habitats.

Through plastic responses, plants adjust processes such as photosynthesis, growth, and resource allocation in response to changing internal or external conditions, improving both individual fitness and population resilience (Morel‐Journel et al., 2020; Verma et al., 2021). At the mechanistic level, plasticity is determined by the interaction between genetic architecture and environmental drivers. The extent of phenotypic flexibility depends on genetic components such as allelic diversity, epigenetic modifications, and regulatory gene networks as well as on external factors like temperature, light, water, and nutrient availability (Morel‐Journel et al., 2020; Valladares et al., 2007). Under severe abiotic stress, flexibility in one trait may decrease because of strong coordination with other traits, reflecting trade‐offs within the adaptive system (Laitinen & Nikoloski, 2024).

From a developmental point of view, Smith‐Gill divided plasticity into developmental conversion, which is genetically controlled and adaptively maintained, and phenotypic modulation, which results from incomplete buffering against environmental variation (Smith‐Gill, 1983). These distinctions indicate that plasticity arises from both genetic regulation and physiological responses, providing a conceptual basis for understanding how plants adjust to environmental changes and internal developmental cues. The concept of canalization, first proposed by Waddington, complements plasticity by describing the capacity of an organism to maintain a stable phenotype despite genetic or environmental variation (Waddington, 1942). Later studies defined canalization as a heritable buffering process that stabilizes phenotypes (Waddington, 1961, 2014; Wilkins, 1997). Molecular evidence has shown that the loss of the molecular chaperone Hsp90 in *Drosophila* and Arabidopsis reveals hidden genetic variation and increases phenotypic variability (Rutherford & Lindquist, 1998; Sangster et al., 2008). Together, plasticity and canalization represent two complementary strategies, flexibility versus stability, that jointly shape the adaptive potential of plants. Indeed, both concepts have independently been suggested as important breeding strategies for ensuring crop security (Carena, 2011; Duvick, 2001), although recent opinion suggests that at a global level a bet‐hedging strategy in which both robust and plastic breeding strategies are adopted (Alseekh et al., 2025). Importantly, a major mechanistic basis of phenotypic plasticity lies in the ability of plants to rapidly and reversibly reprogram metabolism, which is the focus of this review.

### Metabolic flexibility as a core component of phenotypic plasticity in plants

Among the multiple layers that contribute to phenotypic plasticity, metabolism represents a particularly dynamic system subject to integrative regulation (Gupta & Dutta, 2011; Shaar‐Moshe et al., 2019). Under stress conditions, shifts in metabolite profiles provide a direct readout of cellular physiological status and biochemical acclimation. These metabolic outputs are shaped by hierarchical regulatory networks in which transcription factors coordinate the expression of downstream genes encoding key biosynthetic and catabolic enzymes (Liu et al., 2015; Patra et al., 2013; Price et al., 2004; Saibo et al., 2009; Yang et al., 2022). Operating at the nexus of genotype and phenotype, metabolic networks integrate genetic programs with environmental cues to generate biochemical states that ultimately govern cellular and physiological functions (Matias Rodrigues & Wagner, 2009; Zhan et al., 2022). Indeed, metabolic reprogramming often precedes morphological change (Delker et al., 2022; Duarte et al., 2021). Unlike morphological or developmental adjustments, metabolic responses are typically rapid, reversible, and energetically efficient, enabling organisms to buffer environmental perturbations on short time scales (Kleessen et al., 2014; Seebacher & Beaman, 2022; Sulpice et al., 2013). In plants exposed to combined abiotic stresses, such metabolic and phenotypic plasticity emerges from coordinated, system‐level reconfiguration across interconnected pathways, often involving trade‐offs in carbon allocation and energy balance (Shaar‐Moshe et al., 2019). Such network‐level flexibility allows organisms to maintain functional stability while operating under fluctuating environmental constraints.

In plants, metabolic flexibility is particularly critical as environmental fluctuations are so frequent, multidimensional, and often unpredictable (Knudsen et al., 2018). Given that plants cannot escape unfavorable conditions, adaptive success relies heavily on the capacity to rapidly reprogram metabolic processes *in situ*. Through flexible metabolic adjustments, plants can sustain growth, reproduction, and survival across diverse environments, rendering metabolism the primary physiological buffering system underlying plant phenotypic plasticity.

In this review, we focus on carbon, nitrogen, photorespiratory, and lipid metabolism because these interconnected metabolic systems represent major determinants of plant resource allocation, metabolic homeostasis, and environmental adaptation. Carbon and nitrogen metabolism govern the acquisition and utilization of the principal elemental resources required for growth, while photorespiration provides a key interface coordinating carbon–nitrogen balance and cellular redox status. Lipid metabolism complements these processes through dynamic regulation of membrane properties, energy storage, and lipid‐derived signaling. Together, these metabolic domains provide a representative framework for understanding how plants reorganize metabolism in response to environmental change.

Importantly, metabolic plasticity varies substantially across genotypes and can be both heritable and, to some extent, predictable (Tong et al., 2023). Recent evidence indicates that stress‐responsive metabolic trajectories can be inferred from genomic and epigenomic variation and may reflect the evolutionary histories of plant genotypes in their native environments (Leung et al., 2023; Weiszmann et al., 2023). Despite extensive experimental evidence for metabolic plasticity, integrating these responses across scales and environments remains a major challenge. In this context, computational and mathematical modeling approaches are increasingly recognized as essential tools for capturing dynamic metabolic reprogramming, predicting system‐level behavior, and bridging molecular mechanisms with phenotypic outcomes (Tong et al., 2023).

### How to study the genetic basis of metabolic plasticity

The study of the genetic basis of metabolic plasticity in a given species requires the availability of metabolic profiles from a population of genotypes (e.g. diversity panels, introgression lines) over multiple environments. The environments can be characterized by a gradient in a single or few cues or can be specified by the environment and management (E × M) combination (even in the absence of environmental covariates) (Figure 1a). As a result, the change in a measured metabolite level, or any trait, over the studied environments can be specified by a reaction norm, facilitating both quantification as well as visualization of differences in responses between genotypes across environments (Schneider et al., 2026) (Figure 1b). The quantification of plasticity for each genotype in the population can be conducted in several ways, including nominal and relative measures (Laitinen & Nikoloski, 2019). For instance, nominal measures include multiple plasticity indices, that do not depend on the population used, while relative measures are represented by classical approaches for scoring of plasticity in the context of a given population (e.g. see Finlay‐Wilkinson regression; Finlay & Wilkinson, 1963) as a prime representative or random regression models (Arnold et al., 2019). The resulting quantification for plasticity can be used with quantitative trait loci (QTL) mapping and genome wide association studies (GWAS) approaches, to get insights into the genetic architecture of this heritable trait or predict it using genomic prediction from genetic markers (Kusmec et al., 2018; Liu et al., 2020).

**Figure 1:**
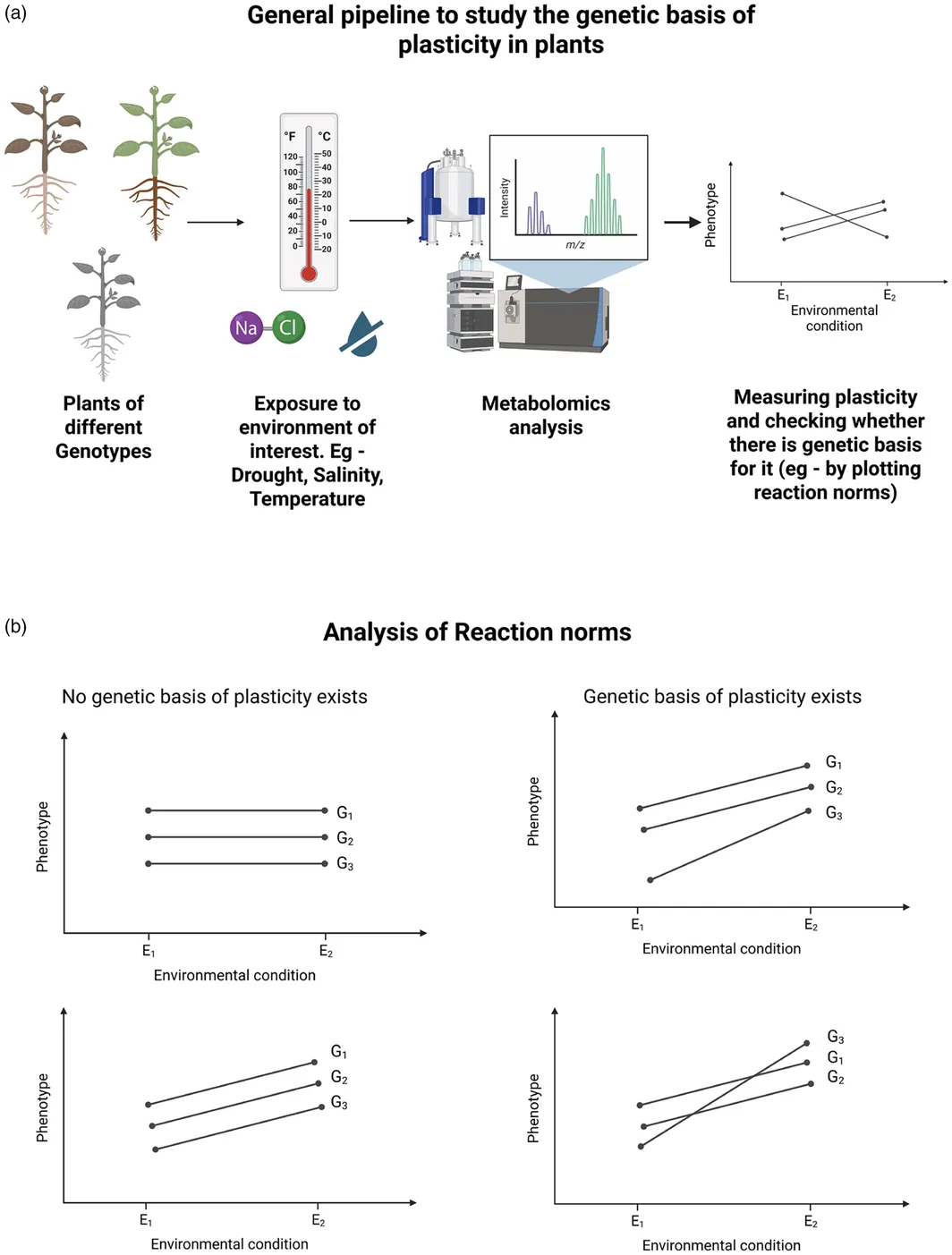
Analysis of plasticity in plants. (a) A general pipeline for deciphering the existence of gene versus environment (GXE) in plants: Plants of different genotypes (which can be sourced from natural ecotypes or introgression lines, for example) are exposed to environmental conditions of interest in different magnitudes. Following this, metabolites of interest are measured using mass spectrometry, following which reaction norms are plotted for each genotype—which detail the phenotype (in our case metabolite concentrations) trend with each environmental condition. Created with BioRender.com. (b) An overview of how reaction norms can be interpreted: Reaction norms tell us two things—one is the existence of GXE—which can be deciphered by determining whether they are parallel to one another or not, where the former signifies its absence while the latter signifies its presence. In the absence of GXE (Panel (i)), one checks whether there is a change in the phenotype with change in the environment. Panel (i), top shows a case where this does not happen—which shows a lack of plasticity of the given trait in the under the environmental condition in question, whereas Panel (ii), bottom shows a presence of plasticity of the trait in question under the given environment as shown by change in phenotype with environment, but no GXE. When GXE is present (Panel (ii)), one can see whether the ranking of the phenotype in any of the genotype changes under a given environmental condition (i.e., whether the reaction norms for the different genotypes crisscross each other). If they do not, the GXE is called ‘rank‐preserving’ (Panel (ii)—top), whereas if they do, the GXE is said to be ‘non‐rank‐preserving’ (Panel (ii)—bottom). Created with BioRender.com.

Taken together, the above considerations highlight metabolic plasticity as a central mechanism by which plants balance robustness and flexibility in fluctuating environments. In this review, we synthesize current knowledge on plasticity in primary and lipid metabolism, with a particular focus on how environmental cues, genetic perturbations, and genotype‐by‐environment interactions shape nitrogen and lipid metabolic plasticity in plants.

## PLASTICITY OF CARBON METABOLISM IN PLANTS

### Metabolic strategies for carbon allocation and storage

Carbon (C) metabolism is a crucial process by which plants accumulate and utilize carbon for growth and survival. In photosynthetic tissues, carbon fixation via the Calvin–Benson cycle occurs during daylight, allowing the synthesis of sucrose through a sequence of reactions. Sucrose is then transported to sink tissues to support growth or for long‐term storage, whereas a considerable amount of the fixed carbon is stored as transitory starch in the chloroplast (Apriyanto et al., 2022). In the dark phase and/or stress conditions, this transitory starch is catabolized by the concerted action of several degradative enzymes to maltose and glucose, which are exported by the maltose transporter and glucose translocator, respectively (MacNeill et al., 2017).

In carbon metabolism, plasticity allows plants to adjust how they store carbon depending on their conditions (Fernie & Tohge, 2013). Carbohydrate plasticity is crucial for phenotypic plasticity in plants, enabling them to adapt to environmental stresses such as nutrient deficiency, drought, and soil compaction. Plants dynamically allocate carbon to roots or shoots based on resource availability, which can enhance their fitness in specific environments. In nutrient‐deficient conditions, plants allocate more carbon to root growth to enhance nutrient acquisition, provided assimilates are not limiting (Ericsson, 1995; Poorter & Nagel, 2000). This alteration in carbon allocation is considered an adaptive response that prioritizes the capture of below‐ground resources.

Different plants have developed different ways to store carbon, which shows their flexibility in adapting to different environments (Fettke & Fernie, 2025). While starch is the most common form of carbon storage in major crops such as grasses and tubers (Piperno et al., 2004; Zierer et al., 2021), several metabolic innovations have led plants to evolve alternative carbon storage strategies. These include simple sugars such as sucrose and hexoses (Rodrigues et al., 2020; Centeno et al., 2011), organic acids such as malate and fumarate (Zell et al., 2010), and more specialized molecules such as fructans (Van den Ende, 2022; Versluys et al., 2018) and raffinose family oligosaccharides or raffinose oligosachharide (RFOs) (Yan et al., 2022). Fructans are special carbohydrate polymers that only certain plant groups can synthesize (Versluys et al., 2018). There is variation in fructan types produced by different species, reflecting on metabolic diversification within this phylogenetically restricted group. Chicory (*Cichorium intybus*) and artichoke (*Cynara cardunculus*) contain inulin, while fodder grasses such as *Dactylis glomerata* and *Phleum pratense* contain levan‐type fructans (Van den Ende, 2022; Versluys et al., 2018). These plants typically grow in cold, dry places, suggesting that fructans help protect against cold and drought (Versluys et al., 2018). Unlike insoluble, plastid‐bound starch, fructans are soluble and vacuole‐localized (Ende, 2013; Valluru & Van den Ende, 2008), allowing them to directly lower cell osmotic potential and depress the freezing point of cell sap, reducing freeze‐induced plasmolysis (Livingston III et al., 2009). They also stabilize membrane phospholipid bilayers by inserting between polar lipid headgroups, limiting solute leakage during freeze‐induced dehydration (Vereyken et al., 2001, 2003)—a mechanism unavailable to starch—and can be mobilized into osmotically active sugars faster, since they bypass the plastid‐hydrolysis and export steps starch requires. Together, these properties likely explain the association of fructan‐accumulating species like *C. intybus* and *C. cardunculus* with cold, dry habitats, relative to starch‐storing relatives (Van den Ende, 2022; Versluys et al., 2018). Similarly, RFOs have been implicated in long‐distance transport processes that contribute to heat and biotic stress tolerance (Yan et al., 2022), further underscoring the metabolic plasticity underlying plant stress responses.

Tomatoes (*Solanum lycopersicum*) store varying amounts of sucrose and hexoses depending on the variety (Osorio et al., 2014). Sugar storage is also important in crop plants like sugarcane (*Saccharum* spp.) and sugar beet (*Beta vulgaris*), which store massive amounts of sucrose (Healey et al., 2024; Rodrigues et al., 2020). Studies show that these differences in sugar storage come mainly from allelic variations in existing genes rather than from the evolution of completely new genes (Alseekh et al., 2021; Fridman et al., 2004). These studies establish a foundation to assess how natural variation in sugar levels contributes to plant phenotypic plasticity under varying environmental conditions. In addition to sucrose, sugar alcohols such as sorbitol accumulate in fruits like apple, functioning as compatible solutes that support osmotic adjustment without disrupting enzyme activity at high concentrations and providing additional protection through reactive‐oxygen‐species scavenging (Kanayama, 2009).

Furthermore, all plants determine defense levels and recruit appropriate outputs against bacterial and fungal pathogens by monitoring sugar levels. Perception of bacterial flagellin activates sugar transporters that increase the cytosolic glucose‐6‐phosphate, that signals through hexokinase to initiate and promote salicylic acid synthesis for antibacterial immunity (Yamada & Mine, 2024). The starch‐deficient phosphoglucomutase (*pgm*) mutant in Arabidopsis exhibits an impaired penetration resistance against the hemibiotrophic fungus *Colletotrichum higginsianum*, and key starch enzymes like the AGPase, BAM1, AMY3 are regulated via redox reactions by thioredoxins, providing a direct mechanistic relation between pathogen triggered reactive oxygen species (ROS) release and altered starch turnover (Skryhan et al., 2018). Together, these findings reveal that starch metabolism is manipulated by the pathogens for it to secure nutrients and suppress defense, while plants mobilize it to power immunity and generate signaling molecules that calibrate the strength and type of their response.

Organic acids such as fumarate and malate also serve as carbon stores, though this role was discovered more recently (Chia et al., 2000; Zell et al., 2010). Fumarate and malate accumulate diurnally in a pattern resembling starch and sucrose, acting as transient carbon stores in species such as soybean and sunflower (Chia et al., 2000). Unlike starch, these organic acids are interconverted by single enzymatic steps (fumarase, malate dehydrogenase), enabling rapid metabolic buffering under cold, and malate additionally couples to redox shuttling between the chloroplast, mitochondrion, and cytosol, balancing the adenosine triphosphate/nicotinamide adenine dinucleotide phosphate (ATP/NADPH) ratio (Weiszmann et al., 2023; Zell et al., 2010). Notably, cytosolic fumarate accumulation via FUM2 is required for photosynthetic acclimation to low temperature in Arabidopsis, indicating a signaling role beyond passive carbon storage (Dyson et al., 2016). Malate is particularly important in the 33 plant families that employ crassulacean acid metabolism (Heyduk, 2022).

Plasticity in another important storage carbohydrate store—that of starch has been received recently (Fettke & Fernie, 2025). However, for completeness, please see the Supplementary Text.

These environmentally responsive changes in carbon allocation cannot be considered independently of nitrogen metabolism, because carbon skeletons and energy generated through carbon metabolism directly support nitrogen uptake and assimilation. This close metabolic coupling provides the basis for considering nitrogen metabolism as a second major component of plant metabolic plasticity.

## PLASTICITY IN NITROGEN METABOLISM IN PLANTS

Nitrogen (N) metabolism comprises a series of coordinated processes involved in the uptake, transport, assimilation, and remobilization of inorganic and organic N, including nitrate (NO_3_^−^) and ammonium (NH_4_^+^) acquisition, nitrate reduction, ammonium assimilation, amino acid biosynthesis, and N translocation within plants (Figure 2b) (Schjoerring et al., 2000, 2006). As a central component of primary metabolism, N metabolism provides the biochemical basis for plant growth and biomass accumulation while serving as a key metabolic hub linking environmental signals to physiological responses (Foyer et al., 2001). Nitrogen metabolism exhibits pronounced plasticity under changing environmental conditions and genetic backgrounds through dynamic regulation of N uptake, assimilation, transport, and allocation (Figure 2a,b) (Acosta‐Motos et al., 2017; Fahad et al., 2017; Florian et al., 2013; Hungria & Kaschuk, 2014; Kaiser & Huber, 2001; Léran et al., 2014; Zayed et al., 2023). At the population level, natural genetic variation further contributes to diversity in N acquisition, utilization, and stress responsiveness, providing an important basis for understanding N‐metabolic plasticity in plants (Bascuñán‐Godoy et al., 2018; Hafeez et al., 2023).

**Figure 2:**
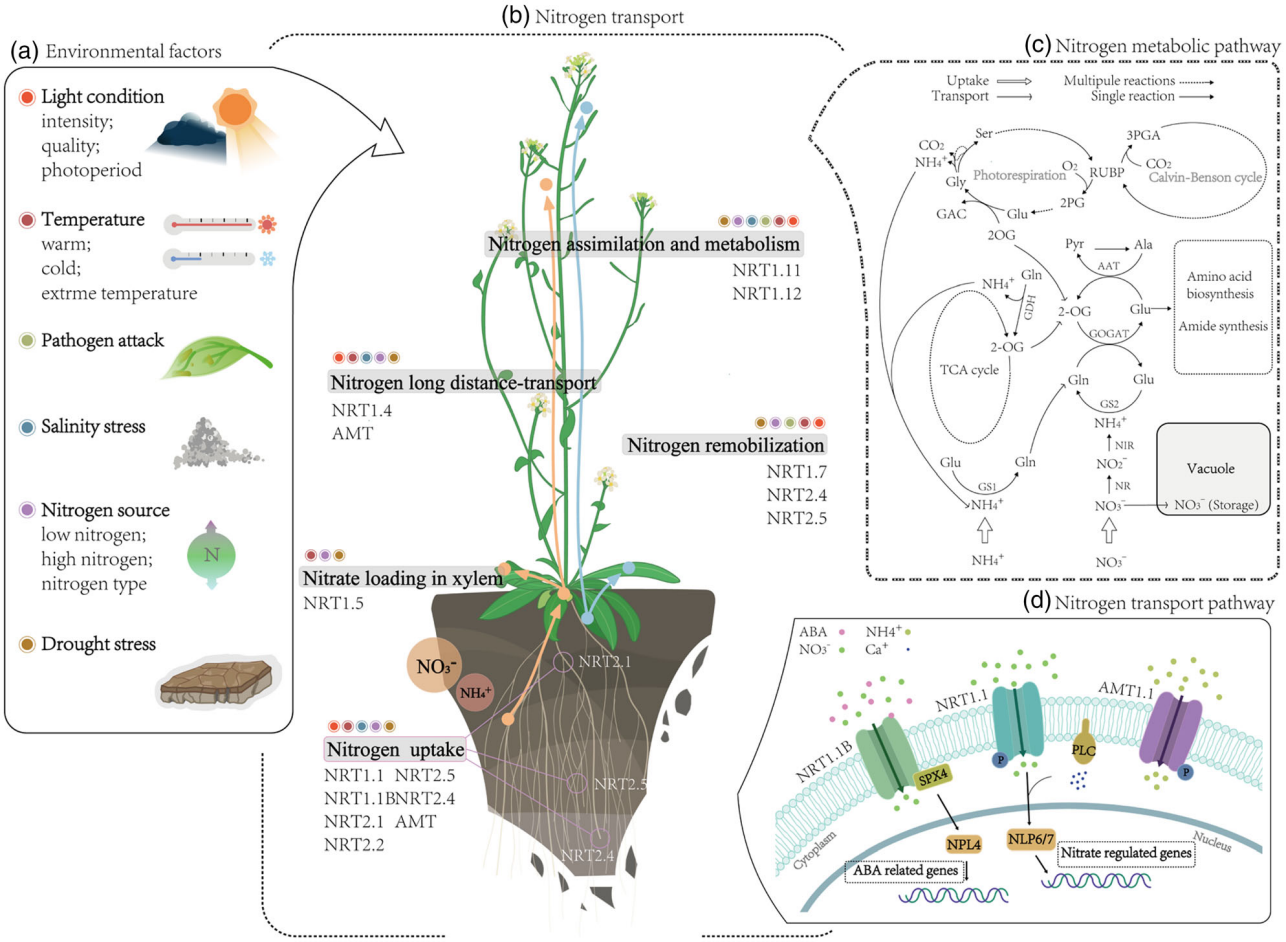
Overview of the plant nitrogen metabolism system under different environments. (a) Environmental factors: Colored circles within each ring represent distinct types of environmental factors. (b) Nitrogen transport: (Yellow route) Plant nitrogen metabolism is initiated by nitrogen uptake in roots. Nitrogen is subsequently transported to the xylem at the root–shoot junction and distributed to aboveground organs. Within cells, nitrogen undergoes assimilation and is incorporated into various metabolic compounds. (Blue route) In addition, plants dynamically regulate nitrogen allocation at different developmental stages. For example, proteins and amino acids in senescing leaves are degraded and remobilized to young leaves and reproductive organs for reassimilation and metabolic conversion. Circles corresponding to those in section a indicate that these environmental factors exert significant influences on this process. (c) Nitrogen metabolic pathways: Schematic representation of nitrogen assimilation (glutamine synthetase [GS]/GOGAT cycle) and the major routes of nitrogen transformation within metabolic networks. (d) Regulation and transport mechanisms: Fundamental mechanisms and pathways governing nitrogen transport and regulation in plants.

### Nitrogen metabolic plasticity under different environmental stresses

#### Nitrogen source

Nitrogen deficiency is one of the most common nutritional constraints affecting N metabolism (Fan et al., 2017; Naz et al., 2019). Reduced N availability limits N uptake and assimilation, decreases the abundance of key N‐containing metabolites such as glutamine, aspartate, and asparagine, promotes soluble sugar accumulation, and induces large‐scale metabolic reprogramming (Xue et al., 2024; Zhao et al., 2018). The extent of these metabolic changes varies considerably among genotypes. N‐efficient genotypes generally maintain higher basal levels of amino acids and related metabolites, while glycerate‐3‐phosphate, valine, *O*‐acetylserine, asparagine, and *N*‐acetylserine are consistently enriched under N‐sufficient conditions, indicating pre‐existing metabolic configurations favorable for N utilization (Huang et al., 2008; Obata & Fernie, 2012; Yoneyama & Kumazawa, 1975). Excess nitrogen also reprograms N metabolism in a dose‐dependent manner. While moderate N supply promotes N assimilation and protein synthesis, excessive N disrupts metabolic balance, reduces nitrogen‐use efficiency, and alters N partitioning, indicating that metabolic responses to elevated N are not always beneficial (Feng et al., 2024; Rossini et al., 2011; Wu et al., 2023; Zhou et al., 2020).

In addition to N availability, N source identity is a major determinant of N‐metabolic plasticity. Although nitrate is the predominant N source for most species, ammonium is energetically advantageous because it can be directly incorporated into organic metabolism without prior reduction (Pelissier et al., 2024). However, excessive or exclusive ammonium nutrition frequently causes physiological stress and metabolic toxicity, requiring tight regulation of ammonium uptake and assimilation (Bittsanszky et al., 2015; Britto & Kronzucker, 2013; Hachiya & Sakakibara, 2017; Kronzucker et al., 2001). Variation in ammonium tolerance among species and genotypes largely reflects differences in ammonium transport, assimilation capacity, and coordination between carbon and nitrogen metabolism (Pelissier et al., 2024).

#### Light

Under high or optimal light conditions, enhanced photosynthetic electron transport increases the availability of ATP, NADH/NADPH, and reduced ferredoxin, thereby promoting nitrate reduction, ammonium assimilation, and the activities of nitrate reductase (NR), nitrite reductase (NIR), and the chloroplast‐localized glutamine synthetase (GS)/GOGAT (Glutamine Oxoglutarate Aminotransferase) cycle, ultimately supporting amino acid biosynthesis, growth, and biomass accumulation (Hachiya et al., 2021; Kramer et al., 2022). Conversely, under low‐light or shaded conditions, reduced photosynthetic electron transport limits the availability of ATP, NADH/NADPH, and reduced ferredoxin, thereby constraining nitrate reduction, ammonium assimilation, and the activities of NR, NIR, and the GS/GOGAT cycle; this in turn suppresses nitrate uptake, amino acid biosynthesis, and ultimately growth and biomass accumulation (Hachiya et al., 2021; Kaiser & Huber, 2001). Concurrently, N metabolism is reoriented toward internal recycling and remobilization, with greater reliance on cytosolic ammonium assimilation and amino acid turnover pathways (Vernizzi et al., 2020).

#### Temperature stress

Temperature is a major environmental factor regulating plant growth and metabolism, and temperatures outside species‐specific optimal ranges impose heat or cold stress that constrains plant development (Ding et al., 2024; Wahid et al., 2007). Plant responses to thermal stress vary with species, developmental stage, and stress intensity and duration (Christophe et al., 2011).

Heat stress disrupts protein stability, membrane integrity, and cellular metabolism, thereby affecting both vegetative and reproductive growth (Ruelland & Zachowski, 2010). Although moderate increases in temperature may enhance biological nitrogen fixation in some species, sustained or severe heat stress generally suppresses N metabolism by inhibiting root growth, nitrate uptake, and N assimilation, resulting in reduced N accumulation, remobilization, and whole‐plant N fluxes (Akhtar et al., 2024; Blais et al., 2012; Sathya et al., 2017). Heat‐induced accumulation of ROS further disrupts cellular homeostasis and contributes to metabolic dysfunction (Samat et al., 2025). Low‐temperature stress, including chilling and freezing stress, impairs photosynthesis, carbohydrate metabolism, protein folding, ROS scavenging, and membrane stability, while freezing additionally causes cellular dehydration and membrane damage through ice crystal formation (Ding et al., 2024; Feng et al., 2025). These physiological disturbances collectively suppress N uptake, transport, assimilation, and remobilization, leading to reduced N‐metabolic fluxes (Fürtauer et al., 2019; Gong et al., 2026).

Plants exposed to non‐lethal temperature extremes can acquire enhanced tolerance through heat or cold acclimation, which involves coordinated physiological, biochemical, and molecular adjustments (Thomashow, 1999). During cold acclimation, accumulation of osmoprotective amino acids and N‐containing compounds, including proline, glycine betaine, and glutamine, contributes to ROS detoxification and maintenance of N metabolism (Ding et al., 2019; Hassan et al., 2020; Kaplan & Guy, 2004; Lardos et al., 2024; Steponkus et al., 1998). Similarly, thermal acclimation can alter plant N status and C/N balance, linking N metabolism to adjustments in photosynthetic and respiratory processes (Way & Sage, 2008).

#### Salinity stress

Salinity stress profoundly disrupts N metabolism by affecting nitrate and ammonium uptake, assimilation, and allocation within plants (Arghavani et al., 2017; Mohamed, 2012; Rais et al., 2013; Zaki, 2016). Under saline conditions, osmotic stress, membrane destabilization, and ionic competition collectively restrict NO_3_^−^ and NH_4_^+^ acquisition, impair nutrient transport, and suppress photosynthesis and plant growth (Acosta‐Motos et al., 2017; Ahmad et al., 2013; Ashraf et al., 2017; Khan et al., 2014). In particular, competition between Cl^−^ and NO_3_^−^ is a major factor limiting nitrate uptake and long‐distance transport, thereby reducing overall N acquisition (Abdelgadir et al., 2005; Annunziata et al., 2016; Hütsch et al., 2016).

Salinity further constrains N assimilation by inhibiting nitrate transporter activity, reducing water‐driven nitrate uptake, and suppressing key N‐assimilatory enzymes (Ehlting et al., 2007; Queiroz et al., 2012; Balliu et al., 2015). NR is particularly sensitive to salinity, and its inhibition limits NH_3_ production for amino acid biosynthesis via the GS/GOGAT pathway (Kaiser et al., 2002; Wang, Wu, et al., 2012). Meanwhile, enhanced protein hydrolysis frequently promotes intracellular NH_4_^+^ accumulation, further exacerbating N‐metabolic imbalance (Wang et al., 2008).

#### Drought stress

Drought stress markedly constrains N metabolism by reducing soil water availability and transpiration‐driven mass flow, thereby limiting N uptake, transport, assimilation, and remobilization (Ahanger et al., 2021; Fahad et al., 2017; Hussain et al., 2018). Because both NH_4_^+^ and NO_3_^−^ must dissolve in soil solution before uptake, drought‐induced reductions in water mobility restrict the acquisition of both N forms, although NO_3_^−^ uptake and assimilation are generally more strongly affected (Alet et al., 2012). Consequently, drought is often associated with relative NH_4_^+^ accumulation and reduced nitrate assimilation, with the activities of NR and NIR representing particularly vulnerable steps in the N‐assimilation pathway (Rennenberg et al., 2006).

Beyond restricting N availability, drought induces extensive transcriptional and enzymatic reprogramming of the N‐metabolic network. Altered expression of N uptake‐ and assimilation‐related genes, together with reductions in the activities of key enzymes such as NR and GS, limits the conversion of inorganic N into amino acids and proteins and ultimately reduces N‐metabolic fluxes (Huang, Li, Shao, et al., 2018; Huang, Li, Zhou, et al., 2018; Robredo et al., 2011). Interestingly, when leaf N status is maintained at relatively high levels, stomatal sensitivity to drought is enhanced, facilitating the maintenance of higher photosynthetic performance and partially buffering the negative effects of drought on N metabolism (Meng, Su, et al., 2016; Meng, Zhang, et al., 2016).

#### Biotic stress

Pathogen infection triggers extensive but nuanced remodeling of nitrogen‐containing metabolites. Although the overall amino acid pool often increases, aromatic amino acids associated with defense‐related secondary metabolism accumulate, whereas aspartate levels consistently decline, indicating selective reconfiguration of nitrogen fluxes rather than uniform metabolic suppression (Berger et al., 2020). Nitrogen metabolism exhibits both conserved and pathogen‐specific responses across bacterial and fungal infections (Ding et al., 2021; Wang, Gu, et al., 2018). Coordinated reorganization of primary and secondary metabolism, including N metabolism, enables plants to dynamically balance growth, defense, and survival during pathogen infection (Zrimec et al., 2025).

### Nitrogen metabolic plasticity under genetic perturbation

Plants acquire nitrogen primarily in the form of nitrate and ammonium, whose availability is determined by microbially mediated soil N transformations (Akhtar et al., 2024; Dodds & Whiles, 2010; Zayed et al., 2023). Once absorbed, nitrate is reduced to nitrite and subsequently ammonium through the sequential action of NR and NIR, after which ammonium is assimilated via the GS–GOGAT pathway. This process generates glutamine and glutamate, the major N carriers that support amino acid biosynthesis, N redistribution, and the synthesis of diverse N‐containing metabolites (Figure 2c) (Masclaux‐Daubresse et al., 2006; Schjoerring et al., 2002). As a result, the glutamine/glutamate‐centered assimilation pathway constitutes a highly conserved core of plant N metabolism and provides the molecular framework through which genetic perturbations influence N acquisition, assimilation, and utilization (Masclaux‐Daubresse et al., 2010).

#### Nitrogen transport

Nitrate is the predominant inorganic N source absorbed by most terrestrial plants, and its uptake is primarily mediated by members of the NRT1/NPF and NRT2 transporter families (Figure 2b,d) (Houdusse et al., 2007; Zayed et al., 2023). The NRT1/NPF family constitutes a large and functionally diverse transporter group that not only mediates nitrate uptake but also transports hormones, amino acids, peptides, and other metabolites, thereby integrating N acquisition with broader metabolic and developmental processes (Forde, 2000; Iqbal et al., 2020; Léran et al., 2014). In contrast, the NRT2 family functions predominantly in high‐affinity nitrate uptake under N‐limiting conditions, with NRT2.1 and NRT2.2 serving as major uptake components, whereas NRT2.4 and NRT2.5 contribute to nitrate acquisition during prolonged N starvation or in specific tissues (Léran et al., 2014; Wang, Cheng, et al., 2018; Wang, Hsu, & Tsay, 2012).

Among these transporters, NRT1.1 functions as a dual‐affinity nitrate transporter whose activity is dynamically regulated by post‐translational modification in response to N availability, enabling plants to rapidly adjust nitrate uptake across fluctuating environments (Choi et al., 2023). Similarly, members of the NRT2 family require the partner protein NAR2 for full activity, and the NRT2–NAR2 complex represents a central component of the high‐affinity nitrate uptake system (Engelsberger & Schulze, 2012; Kotur et al., 2012; Menz et al., 2016; Yong et al., 2010; Zou et al., 2020).

Following root uptake, nitrate is distributed throughout the plant through a coordinated transport network. NPF7.3 (NRT1.5) participates in xylem loading and root‐to‐shoot nitrate transport (Lin et al., 2008), whereas NPF2.13 (NRT1.7) and NRT2.4 contribute to nitrate remobilization under N starvation, facilitating nitrate transfer from source to sink tissues (Fan et al., 2009). In addition, NPF1.1 (NRT1.12) and NPF1.2 (NRT1.11) mediate nitrate transfer between the xylem and phloem, thereby supporting long‐distance nitrate allocation within the plant (Hsu & Tsay, 2013; Kiba et al., 2012; Krapp et al., 2014).

In addition to nitrate, ammonium is an important inorganic N source for plants and is often preferentially utilized under N‐limited conditions because its assimilation requires less energy than nitrate reduction (Bloom et al., 2002; Bowen & Clark, 1981; Ohlund & Nasholm, 2001). Ammonium transporters (AMTs) constitute the core genetic components controlling NH_4_^+^ acquisition and distribution (Figure 2d). Beyond mediating NH_4_^+^ uptake from the rhizosphere, AMTs also participate in long‐distance N transport, symbiotic N transfer, and disease‐resistance‐related processes, highlighting their broader roles in plant N metabolism (Esmaeilzadeh‐Salestani et al., 2022; Giehl et al., 2017; Wu et al., 2022). However, excessive accumulation of NH_4_^+^ can exert toxic effects on plant growth and development, necessitating tight genetic control over its uptake and internal transport (Bittsanszky et al., 2015; Hachiya et al., 2012; Renard et al., 2004).

#### Regulation of transporters

Nitrogen absorbed by the roots is transported to the aerial parts via the xylem and is preferentially allocated to developing organs with high nitrogen demand but low transpiration rates, such as young leaves and reproductive tissues. Efficient N utilization depends not only on transporter abundance but also on their dynamic regulation in response to changing N availability (Choi et al., 2023). Among nitrate transporters, NRT1.1 (CHL1) functions as a prototypical nitrate transceptor that integrates nitrate sensing, uptake, and signaling. Beyond mediating nitrate transport, NRT1.1 regulates lateral root development through auxin transport and dynamically switches between high‐ and low‐affinity transport modes via post‐translational modification, thereby coupling external nitrate availability with root architecture and transcriptional regulation of N‐responsive genes (Bouguyon et al., 2015; Chattha et al., 2022; Fang et al., 2021; Hu et al., 2015; Izmailov & Nikitin, 2020; Krouk et al., 2010; Maghiaoui et al., 2020; O'Brien et al., 2016; Tsay et al., 2007). Recent studies further identified NRT1.1B as a dual sensor of nitrate and abscisic acid (ABA), linking nitrate, auxin, and ABA signaling through the NRT1.1B–SPX4–NLP4 pathway to coordinate nutrient responses with abiotic stress adaptation (Figure 2d) (Ma et al., 2025).

Regulation of the high‐affinity nitrate uptake system is centered on the NRT2.1–NAR2 complex, whose activity is dynamically controlled by post‐translational modification and C‐terminally encoded peptide‐mediated long‐distance signaling, enabling plants to rapidly adjust nitrate acquisition under fluctuating N conditions (Jacquot et al., 2020; Ohkubo et al., 2017, 2021; Okamoto et al., 2006; Orsel et al., 2006; Wirth et al., 2007; Yong et al., 2010).

Besides that, nitrate signaling is coordinated by a complex regulatory network involving multiple transcription factors, including the NLP, TGA, and LBD families, together with Ca^2+^‐dependent signaling pathways (Vidal et al., 2020). In rice, nitrate‐induced Ca^2+^ influx promotes phosphorylation of OsNLP3, thereby activating the expression of nitrate‐responsive genes (Wang et al., 2026).

#### Transcription factors and miRNAs

In addition to transporter‐mediated regulation, N metabolism is extensively controlled at the transcriptional and post‐transcriptional levels. Among the earliest identified nitrate‐responsive transcription factors, ANR1 regulates nitrate‐dependent lateral root development and mediates adaptive root architectural responses to N availability (Liao et al., 2008; Zhang & Forde, 1998). Other transcription factor families, including GATA, DOF, and WRKY, further coordinate N acquisition, assimilation, and carbon–nitrogen metabolism through transcriptional reprogramming of N‐responsive genes and metabolic pathways (Bi et al., 2005; Yanagisawa et al., 2004; Zuluaga & Sonnante, 2019). Post‐transcriptional regulation provides an additional layer of control over N metabolism. The miR169–NFYA regulatory module responds to N deficiency by modulating the expression of nitrate transporter genes, including NRT2.1 and NRT1.1, thereby regulating N uptake and remobilization. Similar regulatory mechanisms have also been reported in maize and wheat, suggesting evolutionary conservation across plant species (Gao et al., 2019; Heerah et al., 2019; Hu et al., 2018; Lee et al., 2020).

#### The plasticity of nitrogen metabolism under natural genetic variation

Genetic perturbation‐based studies of nitrogen metabolism have, in turn, provided reverse validation of the complex molecular mechanisms underlying nitrogen uptake, transport, and regulation in plants under natural conditions. Likewise, population‐level metabolic studies have provided forward evidence for the plasticity of nitrogen metabolism driven by natural genetic variation, as well as the genetic basis underlying such plasticity under stress conditions. A recent population‐based study systematically examined growth, defense, and metabolic phenotypes of *Arabidopsis thaliana* under different nitrogen sources and concentrations, identifying a large number of genes associated with nitrogen‐responsive traits. Notably, partial overlap was observed between the genetic perturbations underlying growth plasticity and those associated with nitrogen metabolic plasticity within the experimental scope. In contrast, when nitrate and ammonium were supplied as the sole nitrogen sources, GWAS revealed that most identified genes were specifically associated with one nitrogen source, with only a small subset shared between the two (Katz et al., 2022). In addition, another study reported a more detailed characterization of metabolic responses under both environmental and genetic perturbations. Under carbon limitation, nitrogen limitation, and optimal conditions, biomass, starch, protein, and a wide range of primary metabolites were quantified, along with the activities of key enzymes, including NR and glutamate synthase. Principal component analysis revealed distinct metabolic response patterns under low‐carbon and low‐nitrogen conditions, with nitrate assimilation, ammonium assimilation, and organic acid metabolism contributing most prominently. Furthermore, the study explored potential associations among different forms of metabolic phenotypic plasticity (Sulpice et al., 2013). Taken together, metabolic plasticity in plants under environmental and genetic perturbations has been consistently observed and reported. The underlying genetic mechanisms, as well as the relationships among different forms of phenotypic plasticity, are being progressively uncovered, and these insights are increasingly being applied in genetic breeding (Wang et al., 2026). However, the molecular regulatory mechanisms governing metabolic plasticity remain an important direction for future research.

An aspect of high interest with respect to the plasticity of nitrogen metabolism is photorespiration. For details, see the Supplementary Text. However, particularly important in the context of this review, the levels of metabolites involved in photorespiration have been shown to be plastic in response to nitrogen availability in local *A. thaliana* accessions, based on coefficient of variation analysis (Pandey et al., 2019). Some of these observations were further corroborated in a metabolomics‐driven study of global *A. thaliana* accessions (Duarte et al., 2021), which identified a gene underlying the plasticity of early rosette diameter to N availability. These results showed that there is variation in the plasticity of photorespiratory intermediates to changes in N.

## PLASTICITY IN LIPID METABOLISM IN PLANTS

While carbon, nitrogen, and photorespiratory metabolism illustrate how plants reorganize central metabolic fluxes under environmental change, adaptation also requires dynamic adjustment of cellular membranes, energy reserves, and lipid‐derived signaling. Lipid metabolism therefore represents another major layer of metabolic plasticity through which environmental and metabolic cues are translated into structural and signaling responses. In plants, endogenous lipids are structurally diverse and functionally complex. For physiological interpretation, plant cellular lipids are often grouped into four major classes: triacylglycerols (TAGs), phospholipids, galactolipids, and sphingolipids (Suh et al., 2015). These lipid classes collectively support membrane architecture, energy storage, and lipid‐mediated signaling and are therefore tightly linked to plant growth and stress adaptation. Phospholipids represent a major structural matrix of cellular membranes and contribute to membrane identity by defining biophysical properties such as fluidity, curvature, and permeability. Importantly, lipids are not randomly distributed within membranes. Instead, their spatial organization across organelles and membrane domains critically influences membrane structure, protein function, and cellular signaling.

Plant endogenous lipids are synthesized through compartmentalized biosynthetic pathways and are differentially enriched in distinct organelles, where they participate in membrane biogenesis, metabolic energy balance, and signal transduction (Fan et al., 2019; Horvath & Daum, 2013; Zeng & Yao, 2022). Overall, because lipids play central roles in membrane integrity, energy storage, and cellular signaling, their compositional and structural dynamics represent a major component of metabolic plasticity in plants. Both endogenous membrane lipids and extracellular cuticular lipids can be actively remodeled in response to environmental cues, thereby supporting homeostasis and stress acclimation.

### Lipid metabolic plasticity under different environmental stresses

Recent advances in lipidomics, particularly high‐resolution liquid chromatography–tandem mass spectrometry, have enabled quantitative profiling of lipid plasticity at the level of lipid classes, molecular species, and fatty‐acyl chain features (carbon length and degree of unsaturation), providing new opportunities to link lipid remodeling with physiological outcomes (Kehelpannala et al., 2021; Liu et al., 2023; Scholz et al., 2025; Tenenboim et al., 2016). Notably, different ecological drivers, including temperature, light, water availability, and biotic interactions, trigger distinct patterns of lipidome reorganization that reflect the flexibility of the underlying metabolic network. In the following sections, we summarize recent lipidomic‐based evidence for lipid metabolic plasticity under major environmental stresses.

#### Temperature

Temperature is one of the most influential environmental factors shaping plant lipid composition, primarily through its effects on membrane fluidity and lipid phase behavior. Plant membranes therefore act not only as structural targets of thermal stress but also as dynamic sensors that initiate adaptive remodeling of the lipidome (Ermilova, 2020). Consequently, temperature stress often induces extensive remodeling of membrane lipid composition, particularly through changes in fatty acid (FA) unsaturation (Sattari Vayghan et al., 2020; Yu et al., 2021). Temperature gradients across environments can also influence lipid composition at the population level, as shown by latitudinal variation in seed‐oil unsaturation and cultivar‐specific changes in sea buckthorn oils under northern climates (Linder, 2000; Vuorinen et al., 2015). Under cold acclimation, enhanced glycerolipid desaturation helps counterbalance reduced membrane fluidity (Tasseva et al., 2004). In Arabidopsis and Brassica, cold increases desaturation of major phospholipid and galactolipid species (Tasseva et al., 2004; Welti et al., 2002). While phosphatidic acid (PA) likely acts as a central signaling mediator of adaptive lipidome remodeling (Arisz et al., 2013).

By contrast, heat stress often drives membrane remodeling in the opposite direction by reducing membrane lipid unsaturation and promoting saturation, as observed in *Agrostis* and wheat. In *Agrostis stolonifera*, heat stress elevates the proportion of saturated lipids, and heat‐tolerant varieties already display higher baseline levels of saturated lipids at ambient temperature (Larkindale & Huang, 2004). In wheat, heat induces a characteristic decrease in membrane lipid unsaturation, largely attributable to a reduction in 18:3 acyl groups and a relative enrichment of 18:1 (Narayanan et al., 2016). At the same time, accumulation of unsaturated TAGs under heat treatment supports the notion that the TAG pools may serve as sinks for polyunsaturated FAs removed from membrane lipids during heat stress (Higashi et al., 2015; Shiva et al., 2020). Beyond bulk compositional shifts, heat exposure also promotes broader lipidome remodeling, including the accumulation of oxidized phospholipids, sterols, glycolipids, odd‐chain FAs, and TAGs (Narayanan, 2018). Together, these adjustments preserve membrane homeostasis and illustrate how lipid remodeling functions as a central mechanism of thermal adaptation.

#### Light

Light availability and intensity shape lipidome composition primarily through their effects on energy supply and carbon assimilation. Lipidomic analyses show that darkness is associated with more desaturated glycerolipid species, including MGDG, PC, PE, and SQDG, whereas increasing light intensity shifts lipid profiles toward higher saturation (Szymanski et al., 2014). Mechanistically, light does not appear to directly regulate acyl‐chain desaturation (Browse et al., 1981). High light exerts the opposite effect by increasing NADPH availability (Stitt, 1986) and promoting FA synthesis, but darkness strongly suppresses *de novo* FA synthesis (Ohlrogge & Jaworski, 1997) and can reduce leaf wax coverage, TAG accumulation, and biomass production (Luzarowska et al., 2023). Therefore, light modulates lipid metabolism largely by controlling carbon and reductant availability, thereby indirectly shaping lipidome plasticity (Burgos et al., 2011).

#### Water availability

Drought stress significantly perturbs photosynthesis and cellular redox balance, thereby affecting membrane integrity and lipid metabolism. Water deficit increases membrane rigidity, while drought‐induced ROS promote lipid peroxidation and reduce membrane fluidity (Sun et al., 2020). Lipid remodeling under drought includes changes in chloroplast galactolipids and phospholipids, which help stabilize membrane structure and support stress tolerance (Golldack et al., 2014; Zhai et al., 2013). Genotype‐dependent lipidome plasticity is also evident, as tolerant wheat lines exhibit higher levels of PC, PA, and PG relative to sensitive varieties (Djanaguiraman et al., 2020). Beyond structural remodeling, signaling lipids display marked plasticity under drought and salinity. In rice, salt stress results in elevated levels of phosphatidylinositol bisphosphate, PA, and diacylglycerol (DAG) pyrophosphate, implicating phospholipase C and DAG kinase in stress‐induced lipid signaling reprogramming (Darwish et al., 2009). Moreover, lipid‐derived messengers such as jasmonates, synthesized from linolenic acid, integrate with ABA and gibberellin signaling, coordinating stress responses with growth regulation (Wasternack, 2007; Wasternack & Hause, 2013). Collectively, these studies highlight the dual contribution of lipidome plasticity in maintaining membrane stability and regulating lipid‐derived signaling pathways in response to water‐related stresses.

#### Nutrient availability (N and P)

Fluctuations in nitrogen and phosphorus availability represent major drivers of lipidome plasticity by coupling lipid metabolism to elemental homeostasis. Under phosphate starvation, plants reduce phospholipids and replace them with phosphorus‐free galactolipids and sulfolipids; in Arabidopsis and rice, DGDG can substitute for PC and SQDG can partially replace PG (Shimojima et al., 2015; Sun et al., 2020; Verma et al., 2021). In parallel, nitrogen limitation reshapes galactolipid and phospholipid profiles and promotes TAG accumulation in vegetative tissues, consistent with carbon reallocation toward transient lipid storage under constrained growth (Gaude et al., 2007; Mei et al., 2016; Narasimhan et al., 2013). Nutrient starvation further enhances lipophagy, linking lipid turnover to energy supply and membrane rebuilding (Perez‐Perez et al., 2024). Together, N‐ and P‐responsive lipid remodeling exemplifies metabolic plasticity that integrates membrane homeostasis, resource recycling, and lipid‐mediated signaling under nutrient stress.

#### Biotic stress

Lipid metabolism is highly plastic during plant–pathogen interactions. At the whole‐metabolism level, defense activation involves reallocation of resources from growth to defense pathways, and genome‐scale metabolic modeling in potato highlights the systemic metabolic rewiring underlying this trade‐off (Zrimec et al., 2025). At the lipidome level, pathogen invasion induces extensive remodeling, including TAG accumulation and increased lipid‐droplet abundance during immune activation in Arabidopsis (Fernandez‐Santos et al., 2020; Schieferle et al., 2021; Yu et al., 2021). Specific FAs and lipid species also modulate membrane properties and defense signaling; in particular, unsaturated FAs such as 18:2 and 18:3 accumulate during infection and may contribute to jasmonic acid biosynthesis, thereby coupling structural lipid remodeling with lipid‐derived defense signaling (Ali et al., 2022; Hou et al., 2016; Lim et al., 2017; Upchurch, 2008).

### Lipid metabolic plasticity under genetic perturbation

While environmental stimuli trigger significant lipidome changes (Higashi et al., 2015; Yu et al., 2021), the magnitude, direction, and reversibility of these responses are ultimately shaped by genetic programs governing lipid synthesis, desaturation, trafficking, and turnover (Zhao et al., 2021). Dissecting this genetic control is therefore essential for understanding how plants balance membrane stability with adaptive flexibility (Guzha et al., 2023). Analyses of natural accessions and targeted genetic perturbations, including knockouts and overexpression lines reveal substantial genotype‐dependent differences in lipid remodeling capacity under stress (Cordoba et al., 2023; Degenkolbe et al., 2012; Pranneshraj et al., 2022). Moreover, even in the absence of external stress, perturbing lipid‐related genes can lead to pronounced shifts in lipid composition that resemble stress‐induced remodeling, underscoring lipid metabolic plasticity as a genetically encoded property of the plant metabolic system and highlighting the importance of identifying key genetic regulators that underlie this flexibility (Szymanski et al., 2014). Importantly, genetic perturbation studies show that key regulators not only define basal lipid composition but also determine the responsive range of lipidome remodeling under fluctuating environments, thereby shaping membrane properties, energy partitioning, and lipid‐derived signaling.

#### TAG synthesis/catabolic enzymes (DGAT/PDAT/SnRK1/SDP1) and storage‐utilization balance

DGAT (*acyl‐CoA: diacylglycerol acyltransferase*) and PDAT (*phospholipid: diacylglycerol acyltransferase*) catalyze the final acylation of TAG assembly and thereby shape the FA composition as well as the remodeling potential of TAG pools (Bates & Browse, 2011). Mutants deficient in DGAT1 or PDAT1 typically show reduced TAG accumulation and compromised stress tolerance, whereas overexpression enhances resistance to freezing or heat stress (Arisz et al., 2018; Demski et al., 2020; Tan et al., 2018). In Arabidopsis, loss of PDAT1 abolished heat‐induced TAG accumulation and reduced thermotolerance, whereas *tgd1* mutants that constitutively overaccumulate TAGs display enhanced heat resistance (Mueller et al., 2017; Xu et al., 2005). Under cold conditions, *dgat1* mutants exhibit reduced expression of the cold‐response regulator *CBF2* and its downstream targets, accompanied by decreased TAG and a compensatory rise in PA and DAG (Tan et al., 2018). These findings suggest that DGAT1‐mediated conversion of DAG to TAG is crucial for maintaining the TAG‐PA balance required for freezing tolerance. On the catabolic side, the lipid‐droplet lipase SUGAR‐DEPENDENT 1 (SDP1) mediates TAG hydrolysis and channels released FAs into β‐oxidation. Loss‐of‐function *sdp1* mutants accumulate TAGs and display impaired lipid mobilization during germination or energy deprivation, indicating that SDP1‐driven lipolysis supports flexible reallocation of stored lipids in response to changing metabolic demand (Choi et al., 2022; Fan et al., 2014).

At a higher regulatory level, the energy‐sensing kinase SnRK1 integrates carbon status with lipid metabolism by repressing anabolic lipid synthesis and promoting autophagy‐dependent recycling. Perturbation of SnRK1 or its catalytic subunit KIN10 disrupts stress‐induced autophagy and lipid remobilization under nutrient or salt stress (Peixoto & Baena‐Gonzalez, 2022; Soto‐Burgos & Bassham, 2017). Collectively, DGAT/PDAT‐driven TAG formation, SDP1‐mediated degradation, and SnRK1‐based energy regulation define a core framework for the plasticity of TAG storage and utilization.

#### Fatty acid desaturases (FADs) as key regulators of lipid metabolic plasticity

FADs regulate the degree of acyl‐chain unsaturation and thereby represent a central control point of membrane plasticity. In Arabidopsis, ER‐ and plastid‐localized ω‐6 and ω‐3 desaturases tune the balance between mono‐ and polyunsaturated FAs, directly shaping membrane responsiveness (van Meer et al., 2008). Genetic perturbation studies identify FAD‐mediated desaturation as a central control point of lipid metabolic plasticity. Under tunicamycin‐induced endoplasmic reticulum (ER) stress, lipid profiling of *fad2* mutants and overexpressors demonstrated that an appropriate 18:1/18:2 balance is required for ER membrane function and stress tolerance (Nguyen et al., 2019). Likewise, the *fad6* mutant displays reduced salt tolerance and altered Na^+^/K^+^ homeostasis, implicating plastidial ω‐6 desaturation in osmotic‐stress lipid remodeling (Zhang et al., 2012). Temperature‐responsive regulation of the plastidial ω‐3 desaturases (FAD7/FAD8) further illustrates how desaturation flexibility supports thermal adaptation, and a minimal level of FAD‐dependent trienoic FAs is essential for sustained heat tolerance (Routaboul et al., 2012). ACYL‐LIPID DESATURASES (ADS1/ADS2) modulate chloroplast membrane composition during cold adaptation: *ads1* mutants, reduced 18:1 content and lower abundance of 34‐carbon MGDG species together with enhanced freezing tolerance after cold acclimation. Whereas ads2 mutants display reduced levels of 34‐carbon PG and MGDG species, decreased unsaturated FAs (16:1, 16:2, 16:3, and 18:3) and increased saturated FAs (16:0 and 18:0), leading to severe growth inhibition and freezing sensitivity under low temperature (Chen & Thelen, 2013, 2016). Beyond membrane biophysics, FAD‐dependent production of linolenic acid (18:3) feeds into oxylipin and jasmonic acid (JA) Berlin biosynthesis, linking FA desaturation with stress‐induced hormonal signaling and biotic stress responses (Shah, 2005; Wasternack & Hause, 2013). Natural variation in FAD loci further contributes to genotype‐dependent thermal adaptation, supporting FA desaturation as both a mechanistic regulator of membrane plasticity and an evolutionary substrate for lipid metabolic adaptability (Degenkolbe et al., 2012; Menard et al., 2017).

#### Transcription factors and regulatory networks controlling lipid metabolic plasticity

Beyond enzymatic control, lipid metabolic plasticity is shaped by transcriptional networks that connect hormonal signaling, carbon availability, and stress perception, which are translated into coordinated changes in lipid biosynthesis, remodeling, and barrier formation. WRINKLED1 (WRI1) is a central regulator of FA and TAG biosynthesis that directly activates genes involved in glycolysis and *de novo* FA synthesis (Maeo et al., 2009). WRI1 is transcriptionally controlled by upstream regulators LEAFY COTYLEDON1 and 2 (LEC1/LEC2) and is further modulated by sugar availability, hormonal cues, and the energy sensor SnRK1, placing WRI1 at the intersection of carbon status and environmental responsiveness (Kuczynski et al., 2022; Yang et al., 2022; Zhai et al., 2017). Genetic perturbation of WRI1 results in pronounced changes in seed oil content and FA composition, indicating that it defines a major component of lipid biosynthetic capacity under variable environmental conditions (Focks & Benning, 1998; Singer et al., 2016). By contrast, MYB96 exemplifies transcriptional control of protective lipid barriers under drought: as an ABA‐responsive R2R3‐type MYB transcription factor, it activates cuticular wax biosynthesis through genes encoding FA elongation enzymes (e.g., KCS, KCR, ECR), while its homolog MYB94 acts partly redundantly in this process (Lee et al., 2016; Seo et al., 2011; Wang et al., 2020). Networks of bZIP and LAFL transcription factors, including LEC1, LEC2, FUS3, and ABI3, further integrate developmental programs with lipid metabolic plasticity by coordinating FA desaturation, storage‐lipid biosynthesis, and seed maturation (Jia et al., 2013; Mendes et al., 2013; Wang & Perry, 2013; Yang et al., 2022). Together, enzymatic modules, desaturation control, and transcriptional networks form a hierarchical genetic architecture that determines when and to what extent lipid metabolic pathways are engaged under fluctuating environments.

#### Lipid‐derived hormone genes regulate lipid metabolic plasticity

As for the contribution to lipid metabolic plasticity of the lipid‐derived hormone (please see the Supplementary Text).

Collectively, these environmental responses and regulatory mechanisms illustrate the multilayered control of lipid metabolic plasticity in plants, as summarized in Figure 3.

**Figure 3:**
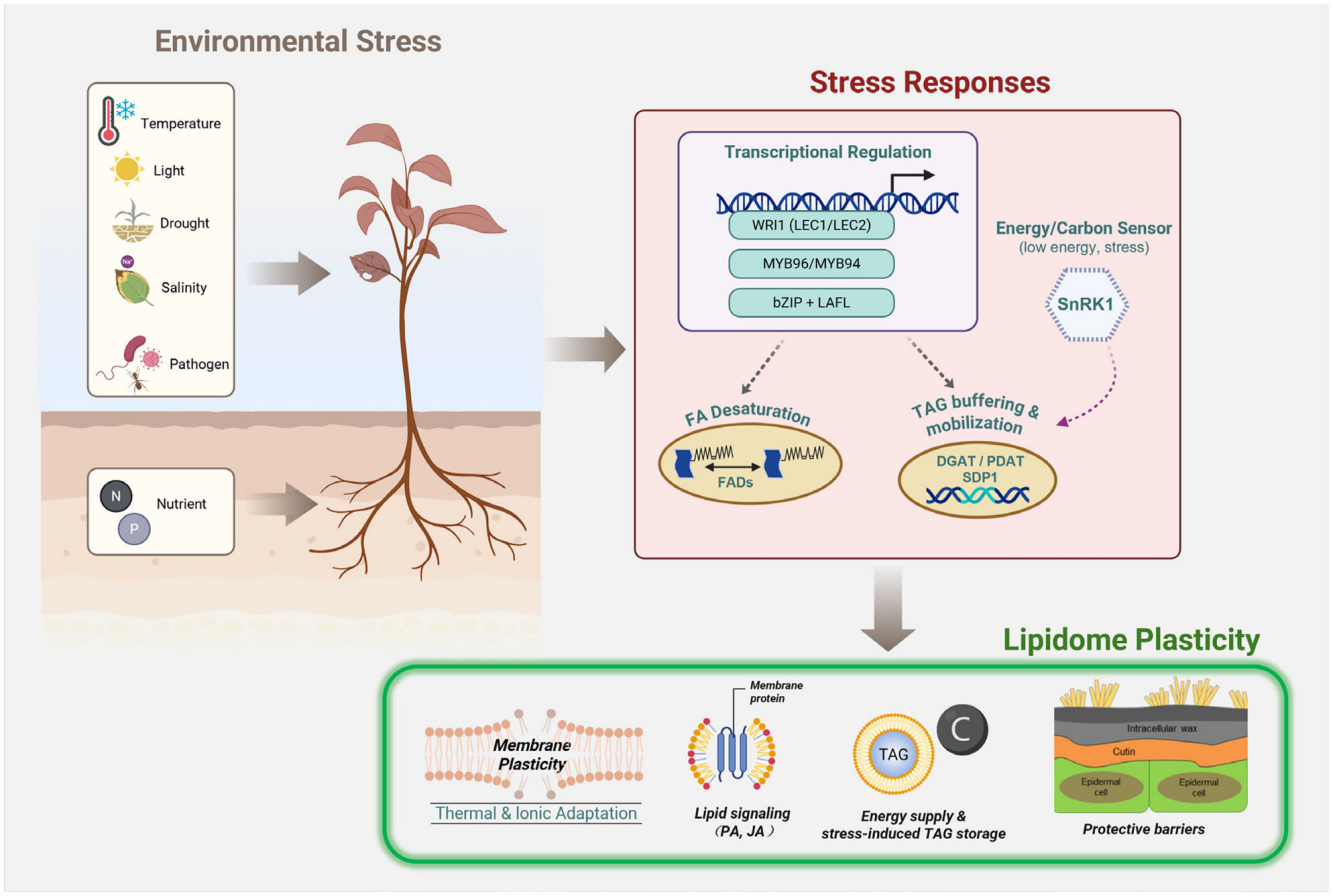
Environmental regulation of lipid metabolic plasticity in plants. Created with BioRender.com. Environmental cues including temperature, light, water availability, salinity, biotic stress, and nutrient status (N and P) act as external drivers that reshape the plant lipidome. These diverse inputs are integrated through a limited set of genetic regulators controlling lipid synthesis, remodeling, storage, and turnover. Key regulatory modules include enzymes mediating triacylglycerol (TAG) synthesis and degradation (DGAT, PDAT, SDP1) under energy control by SnRK1, fatty acid desaturases (FADs) regulating membrane fluidity, and transcriptional regulators such as WRI1, MYB96/MYB94, and bZIP/LAFL factors that define the amplitude and context dependency of lipid metabolic responses. Together, these genetic programs enable dynamic lipidome plasticity, manifested as membrane remodeling, modulation of fatty acid unsaturation, TAG buffering, lipid‐derived signaling, and cuticular lipid formation, thereby supporting plant adaptation to fluctuating environments. Only representative genetic regulators discussed in this review are shown.

### Lipid metabolic plasticity of genetic variation in response to different environmental conditions (G × E)

Metabolic plasticity represents a key molecular process of genotype‐by‐environment (G × E) interactions, reflecting how genetic variation shapes the capacity of plants to reprogram metabolic networks in response to changing environmental conditions. While environmental cues such as temperature, water availability, and biotic stress can bring metabolic responses, the magnitude, direction, and reversibility of these responses vary substantially among genotypes (Kitashova et al., 2023). Such variation highlights that environmental inputs do not solely drive metabolic plasticity but emerge from the interaction between external stimuli and genetically encoded regulatory architectures (Napier et al., 2023). In this context, metabolite profiles—and particularly lipidomic states—serve as sensitive, integrative readouts of G × E interactions, linking genetic variation to physiological performance under stress. Evidence for genetically constrained metabolic plasticity has been most extensively studied in *A. thaliana*, where natural accessions display pronounced differences in metabolic and lipidomic responses to environmental perturbations (Degenkolbe et al., 2012; Weiszmann et al., 2023). Such genotype‐dependent lipidomic responses correlate with variation in freezing tolerance, supporting the idea that lipid metabolic plasticity constitutes an adaptive trait shaped by natural genetic variation.

A major challenge in resolving G × E‐driven metabolic plasticity is to move from descriptive metabolite shifts toward identifying the underlying genetic determinants that modulate metabolic responsiveness across environments. Metabolite‐based genome‐wide association studies (mGWAS) address this gap by treating metabolite abundances (including lipid species) as quantitative traits and associating their variation across genetically diverse panels with genome‐wide polymorphisms. In practice, the power of mGWAS is amplified when paired with high‐resolution metabolomics/lipidomics and experimental designs that explicitly capture environmental perturbations, thereby directly targeting the genetic architecture of metabolic plasticity rather than static metabolite levels (Chen et al., 2020; Luo, 2015; Matsuda et al., 2015).

In *A. thaliana*, association‐based studies have demonstrated how population genetics can be integrated with lipidomic phenotypes to resolve G × E‐driven metabolic plasticity. Analysis of temperature‐dependent seed lipid composition in the Arabidopsis MAGIC population revealed substantial genotype‐specific variation in ω‐6 desaturation and identified FAD2 as a major determinant, directly linking natural genetic variation to lipidomic plasticity under thermal stress (Menard et al., 2017). Beyond static traits, time‐resolved GWAS approaches have proven particularly powerful for capturing dynamic metabolic responses. For example, GWAS conducted across darkness‐induced senescence mapped loci controlling lipid abundances at multiple time points, revealing stage‐ and condition‐specific genetic regulation consistent with G × E‐dependent metabolic control (Zhu et al., 2022). Although not all Arabidopsis mGWAS studies focus exclusively on lipids, large‐scale seed mGWAS frameworks have established robust strategies for mapping metabolite‐to‐locus relationships and reconstructing coherent biochemical pathways, providing methodological templates readily extendable to lipid‐centric analyses (Naake et al., 2024).

Comparable lipidomics‐assisted GWAS approaches in crops further underscore the general relevance of mGWAS for dissecting metabolic plasticity. In maize, GWAS of the leaf lipidome across large, inbred panels uncovered extensive genetic control over diverse lipid species under field conditions, and subsequent analyses leveraged these associations to infer pathway‐level organization of lipid biosynthesis (Li et al., 2019; Naake et al., 2024). In rice, metabolome‐scale GWAS has mapped hundreds of metabolite‐locus associations, yielding genetic and biochemical insights into natural metabolic variation and establishing population resources well suited for lipid‐focused mGWAS under defined environmental treatments (Gong et al., 2013). Collectively, these studies position mGWAS as a powerful bridge between molecular lipid remodeling and population‐scale genetic diversity, enabling the dissection of both static lipid composition and environment‐dependent metabolic trajectories that underline genotype‐specific metabolic plasticity.

Generally, GWAS analyzes phenotypic values measured under a single environmental condition. However, in recent years, studies began to use statistical approaches to quantify the plasticity of specific plant traits across different environments, and these quantified plasticity indices were subsequently used as phenotypic values in GWAS analyses. To date, multiple studies have reported GWAS analyses of phenotypic plasticity in *A. thaliana*, identifying potential regulatory mechanisms and key genes underlying plastic responses (Duarte et al., 2021; Laitinen & Nikoloski, 2019; Pandey et al., 2019). In some cases, these studies have further integrated metabolomic data to characterize the metabolic changes associated with environmental variation. Although the current lack of studies applying mGWAS to metabolic plasticity, such approaches are likely to become an indispensable component of future research on phenotypic plasticity.

### The use of modeling in studying the plasticity of metabolism

Models of plant metabolism enable simulations and predictions of plant metabolic phenotypes (e.g., reaction fluxes, metabolite concentrations) and/or associated physiological outcomes under different environmental conditions. Depending on their type–stoichiometric or kinetic–these models facilitate predictions of steady‐state reaction rates or dynamic reaction fluxes and metabolite concentrations, respectively (Nikoloski et al., 2015), in specifically chosen environments. While stoichiometric models have been assembled for the majority of model plants and crops (Machado et al., 2025), the generation of quantitatively accurate kinetic models is lagging behind (Xu et al., 2026). The challenges lie in the lack of available enzyme parameters (e.g., catalytic rate, kcat, and the related maximal velocity, *V*_max_, as well as Michaelis–Menten constants) and/or data that can be used to improve model parameterization, that entails the determination of values for enzyme parameters. For instance, even in the case of photosynthesis, as the most well‐studied plant‐specific metabolic process, the existing models are parameterized using literature values, estimates from selected data sets, or approximations (Zhu et al., 2013). Even if this issue were resolved for a single genotype of a selected plant species, the study of genetic basis of plasticity necessitates the availability of genotype‐specific metabolic models (see Introduction). These models will facilitate simulations and predictions of metabolic states for individual genotypes under a set of environments, enabling applications of well‐established techniques to dissect the genetic basis of plasticity (Arnold et al., 2019).

To address this issue, recent modeling studies based on stoichiometric models have focused on generation of genotype‐specific stoichiometric models (Tong et al., 2020). To this end, three possible strategies can be followed. First, quantitative metabolic profiles, characterizing the biomass composition of a modeled plant system (e.g., organ or entire rosette), can be used to parameterized a synthetic biomass function that enables modeling of relative growth rate. This strategy was used to obtain metabolic models for 67 *A. thaliana* accessions grown under limiting carbon, limiting nitrogen, and optimal conditions and used them to study the plasticity of fluxes between these growth conditions (Tong et al., 2020). For instance, the study pointed out starch and photorespiratory metabolism as key processes that harbor reactions with notable genotype‐by‐environment variance components of their fluxes. This study also facilitated the first integration of genetic markers to predict fluxes and use them in predicting growth for accessions not used in the training set, demonstrating improvement over the statistical genomic prediction models. The changes in reaction rates between two conditions in the 67 *A. thaliana* accessions were also modeled in terms of genetic markers, demonstrating that predictability of growth in an unseen condition can be further boosted by considering genomic prediction models for plasticity (Tong et al., 2023). This work was expanded to study plasticity of lipid metabolism under different light conditions (Cordoba et al., 2023), enabling the first flux GWAS in plants. Similar idea was used to study the association between fluxes and genetic markers in models of *A. thaliana* and *Populus trichocarpa* (Sarkar & Maranas, 2020). Second, genotype‐specific catalytic rates of enzymes can be used to obtain enzyme‐constrained metabolic models, shown to lead to improved predictions of molecular and physiological traits across different organisms (de Moura Ferreira et al., 2025). While this strategy has not yet been used with different *A. thaliana* accessions, an enzyme‐constrained model was recently parameterized for Col‐0, allowing the study of metabolic reactions and pathways that show particular responses to different temperatures (Wendering et al., 2025). This model together with constraint‐based modeling approaches allowed the identification of genes whose knock‐outs have effects under specific temperatures, which were in turn validated with mutant studies. Together with approaches for parameterization of enzyme‐constrained models (Wendering et al., 2023), this model is expected to contribute to the study of plasticity in enzyme allocation across different genotypes. Third, genotype‐specific models can be instantiated using gene presence‐absence data using pan‐genome‐scale metabolic models (Arend et al., 2025), that is still in its nascent stage for plants.

Parameterization of (large‐scale) kinetic models can be performed using classical parameter estimation approaches (Toumpe et al., 2025), facilitating the generation of model variants based on the availability of genotype‐specific calibration data sets. In the case of plants, genotype‐specific calibration of a kinetic model of C_4_ photosynthesis was recently conducted using photosynthesis response curves for lines from a maize population (Xu et al., 2026). The respective parameters were predicted in terms of genetic markers using genomic predictions, demonstrating a dramatic increase in prediction accuracy of photosynthesis under saturating light for field‐grown unseen lines. Notably, this study demonstrated that the integration of kinetic models with genomic prediction models of enzyme parameters, termed kineticGP, outperformed genomic prediction models that consider genotype‐by‐environment interactions, while providing mechanistic insights into drivers of plasticity in C_4_ photosynthesis. Recent advances in AI have enabled training of neural networks for the purpose of model parameterization (Xu et al., 2026), pushing the boundaries in the development of large‐scale kinetic models of plant metabolism. As a prominent and only example in plants to date, a recent study proposed C4TUNE, an AI‐enabled approach for parameterization of large‐scale kinetic models of C_4_ metabolism. The neural network is trained on sampled parameter values and corresponding photosynthesis response curves and facilitates parameter prediction provided measured photosynthesis response curves as input. Using data from the same maize population, C4TUNE enabled efficient parameterization of genotype‐specific photosynthesis models and, together with *post hoc* analyses, identified parameters that limit photosynthesis. One can easily envision the usage of the predicted parameters with genomic prediction, as in kineticGP, with GWAS approaches to identify loci leading to desired enzyme parameters, as well as with simulations under different environments to identify key factors affecting the plasticity of this important process.

## CONCLUSION

Collectively, the studies reviewed here demonstrate that plant metabolism is highly plastic across multiple major metabolic domains, including carbon, nitrogen, photorespiratory, and lipid metabolism. Although these systems differ in biochemical organization and physiological function, they share a common property: They are dynamically reconfigured in response to environmental variation to maintain growth, survival, and reproduction. In carbon metabolism, plasticity is expressed through flexible allocation and storage strategies, stress‐responsive starch turnover, and diversification of carbon reserve forms. In nitrogen metabolism, plasticity manifests as reorganization of uptake, assimilation, transport, and remobilization processes under changing nitrogen availability and other abiotic and biotic stresses. Photorespiration serves as an integrative node that reshapes the carbon–nitrogen balance under fluctuating environments, while lipid metabolism exhibits pronounced plasticity through membrane remodeling, desaturation, storage–utilization balance, and lipid‐derived signaling. Taken together, current evidence supports the view that metabolic plasticity is not a passive consequence of stress, but a regulated and multilayered property of plant metabolic networks that links environmental sensing with biochemical adaptation.

At the same time, the approaches used to uncover metabolic plasticity are beginning to define a broader roadmap for the field. Population‐scale studies across contrasting nitrogen or temperature environments show that plasticity itself can be treated as a quantitative trait and mapped genetically, while recent work in maize highlights that plastic responses often have complex and distributed genetic architectures rather than simple single‐locus control (Jin et al., 2023). In parallel, reaction‐norm frameworks and more mechanistic modeling approaches provide powerful ways to quantify, interpret, and predict environmentally responsive metabolic phenotypes. Together, these developments suggest that the next phase of research on metabolic plasticity will increasingly depend on integrating large‐scale environmental perturbation experiments, genetic mapping, and predictive modeling into a common framework.

## FUTURE PERSPECTIVES

A major challenge for future research is to move beyond pathway‐specific case studies toward identifying general principles of metabolic plasticity across metabolic systems, environmental contexts, and species. This will require broader investigation of combined, sequential, and fluctuating stresses rather than isolated static treatments, as well as expansion from Arabidopsis and other model systems to diverse natural populations, breeding materials, and crop species. Such work will be essential for determining how metabolic plasticity contributes to agronomic performance and resilience, particularly as recent perspectives on food security emphasize the need to balance plasticity with robustness in crop improvement (Alseekh et al., 2025; Tang et al., 2025). In parallel, modeling is likely to become an increasingly important unifying tool, but current approaches remain limited by incomplete pathway knowledge, insufficient condition‐specific parameterization, and weak representation of regulatory and temporal dynamics. Future progress will therefore depend on tighter integration of genetics, metabolomics, lipidomics, and predictive modeling, with the long‐term aim of explaining and ultimately predicting how plant metabolic networks balance flexibility and stability under environmental change.

## CONFLICT OF INTEREST

None of the authors have a conflict of interest to disclose.

## Supporting information


**Supplementary Text S1.** Plasticity of starch and lipid‐derived hormones.

## Data Availability

Data sharing not applicable to this article as no datasets were generated or analysed during the current study.
