# Supplementary material for: Plasticity of metabolism in plants
Source: Plant J. 2026 Oct 12;128(1):e71159. doi: 10.1111/tpj.71159 (PMC13656763; doi:10.1111/tpj.71159)
Supplement: Supplementary file 1 [file TPJ-128-0-s001.docx]

Enzymatic diversity and plasticity in starch metabolism

Although plants have evolved alternative carbon storage strategies, starch remains the most important carbon storage form in plants. Starch is made of two types of molecules (amylose and amylopectin) with only two types of bonds between sugar units, however, the synthesis and degradation of starch require many different enzymes (Fettke *et al.*, 2005; Apriyanto *et al.*, 2022). Most of these enzymes exist in multiple isoforms within plant genomes (Fernie & Tohge, 2017). These multiple isoforms evolved as a result of plants needing to respond to constantly changing environments, allowing greater flexibility in starch metabolism (Abt & Zeeman, 2020).

Different isoforms can have specialized functions. For example, the enzyme Starch Synthase 4 (SS4) retains the typical enzymatic activity, like other starch synthesis, of transferring glucosyl residues to existing glucan chains at the non-reducing end, but it specifically seems to be involved starch granule initiation (Roldán *et al.*, 2007). Plants lacking SS4 make very few starch granules with only one per chloroplast (Roldán *et al.*, 2007). However, other enzyme mutants also show reduced granule numbers (Malinova *et al.*, 2017; Malinova *et al.*, 2018; Liu *et al.*, 2021; Li *et al.*, 2022), indicating that multiple pathways control how many granules are formed (Mérida & Fettke, 2021; Li *et al.*, 2022).

Similar, starch granule characteristics such as size, morphology, density, and number are controlled by the interplay of synthesizing and degrading enzymes and affect how plants store and use carbon (Liu *et al.*, 2021). Larger granules can store more carbon. The shape matters because enzymes work on the granule surface and flat granules have a greater surface area than round granules of the same volume. Transitory starch that gets synthesized and degraded on a diurnal cycle is usually flat, while long-term storage starch is usually round (Apriyanto *et al.*, 2022). The flat shape of transitory starch provides more surface for enzymes to work on and thus allows a faster turnover.

The important process of starch phosphorylation adds phosphate groups to amylopectin (You *et al.*, 2020; Compart *et al.*, 2023). While scientists originally thought this only happened during starch degradation, it actually also occurs during starch synthesis (Xu *et al.*, 2017; Apriyanto *et al.*, 2022), again pointing to the high flexibility in starch metabolism. Plants without the phosphorylation enzymes make larger, uneven granules (Mahlow *et al.*, 2014; Liu *et al.*, 2021), and phosphorylation also helps in controlling granule size and uniformity (Li et al., 2024).

The plastidial starch phosphorylase (PHS1) is particularly important for controlling granule number and characteristics (Mérida & Fettke, 2021; Seung *et al.*, 2025) . PHS1 can both synthesize and degrade starch molecules (Fettke *et al.*, 2010; Li *et al.*, 2022). Interestingly, wheat endosperm contains large A-type starch granules that initiate during early grain development and small B-type granules that initiate later. Wheat mutants lacking PHS1 produced normal A-type granules but exhibited fewer and larger B-type granules, with the reduction occurring specifically during the B-type granule initiation period at 15-20 days post-anthesis. These findings demonstrate that PHS1 is exclusively required for B-type granule initiation, indicating that A-type and B-type granule initiations proceed through distinct biochemical mechanisms in wheat endosperm (Kamble *et al.*, 2023). In potato, rice, and maize, losing PHS1 changes the internal starch structure and granule size, especially in cold conditions (Koper *et al.*, 2020; Kamble *et al.*, 2023; Sharma *et al.*, 2023), showing that this enzyme becomes more important under certain environmental conditions.

Non-catalytical proteins such as Early Starvation 1 (ESV1) and Like Early Starvation 1 (LESV) alter the granule surface and influence both starch synthesis and degradation (Singh *et al.*, 2022; Liu *et al.*, 2023). Some specific isoforms have lost their ability to synthesize and degrade starch but still play important roles. In Arabidopsis, β-amylase 9 (BAM9) and starch synthase 5 (SS5) are catalytically inactive proteins but play a very important role in regulating starch metabolism by helping guide other enzymes to the right places on starch granules (Abt *et al.*, 2020; David *et al.*, 2022). Several other proteins with no enzyme activity are also essential for normal starch formation (Seung & Smith, 2019), and recent evidence shows that the location where starch initiation occurs inside the chloroplast is not random but controlled by specific proteins (Sharma *et al.*, 2024).

Environmental regulation and phenotypic plasticity

In carbon metabolism, plasticity allows plants to adjust how they store carbon depending on their conditions (Fernie & Tohge, 2013). Carbohydrate plasticity is crucial for phenotypic plasticity in plants, enabling them to adapt to environmental stresses such as nutrient deficiency, drought, and soil compaction. (Ericsson, 1995; Poorter & Nagel, 2000). Under these adverse environmental conditions, plants typically initiate starch remobilization to sustain cellular energy. The soluble sugars and downstream metabolites generated through starch degradation not only support growth under stress but also act as osmoprotectants and compatible solutes, thereby mitigating stress-induced cellular damage and helping maintain osmotic and metabolic homeostasis. (Krasensky & Jonak, 2012; Thalmann & Santelia, 2017). This alteration in carbon allocation is considered an adaptive response that prioritizes the capture of below-ground resources.

Enhanced starch degradation has frequently been associated with improved stress tolerance. For example, in the green alga *Dunaliella tertiolecta*, starch degradation was observed in response to salt stress (Thalmann & Santelia, 2017). Similarly, drought-tolerant cultivars of broad bean (*Phaseolus vulgaris*) exhibit greater starch degradation than drought-sensitive genotypes (González-Cruz & Pastenes, 2012), and in soybean (*Glycine max*), drought-resistant varieties accumulate higher levels of carbohydrates in pods under water deficit (CUELLAR‐ORTIZ *et al.*, 2008). Low temperature also strongly alters photosynthetic capacity and carbohydrate content in plants (Adams III *et al.*, 2016), triggering the accumulation of soluble sugars, a process known as cold-induced sweetening (Herman *et al.*, 2017). These accumulated sugars serve multiple protective functions, including osmoregulation, cryoprotection, cellular signal transduction, and reactive oxygen species (ROS) scavenging. The underlying starch-to-sugar conversion during cold stress is tightly coordinated by heat shock proteins (Hsps), as demonstrated in potato tubers (Lin *et al.*, 2019). In Arabidopsis, cold stress induces coordinated shifts in both the metabolome and the transcriptome; however, transcript levels do not always account for the observed metabolic changes, suggesting that a significant portion of metabolic adjustment occurs through post-transcriptional or post-translational regulation rather than gene expression alone (Kaplan *et al.*, 2007).

Stress-induced starch degradation seems to rely on enzymatic pathways that differ substantially from those operating during normal nocturnal starch turnover. Whereas night-time starch breakdown is predominantly mediated by BAM3 and ISA3, abiotic stress conditions recruit distinct enzymatic combinations. Under water-related stress, for example, AMY3 and BAM1 are strongly induced (Thalmann & Santelia, 2017). The expression of BAM1 is particularly responsive to environmental perturbations: it is upregulated by temperature stress, osmotic stress, and salinity stress, with elevated transcript and protein levels observed in both leaf guard cells and root. These findings indicate that BAM1 plays a central role in stress-activated starch mobilization, functioning independently of the canonical nocturnal starch degradation machinery. Plants deficient in the α-glucan phosphorylase PHS1 also show heightened sensitivity to drought and salinity (Ribeiro *et al.*, 2022). Collectively, these observations so far demonstrate that stress-induced starch degradation is mediated primarily by BAM1, AMY3, and PHS1, the enzymes that are thought to be not so important for normal night-time starch metabolism but seems to be crucial under abiotic stress conditions.

While abiotic stresses generally promote starch degradation, biotic stresses often alter starch metabolism in distinct ways depending on the type of pathogen involved. The molecular mechanisms that cause starch to accumulate under biotic stresses are largely unknown (Ribeiro *et al.*, 2022). The corn smut fungus *Ustilago maydis* deploys the effector Hypertrophy-Associated Protein 1 (Hap1) to inhibit the central metabolic sensor Sucrose Non-Fermenting 1-Related Kinase 1 (SnRK1) in maize, which normally represses starch synthesis and activates defence. Thereby, simultaneously promoting starch accumulation and suppressing immunity (Lee *et al.*, 2026) . Similarly, the citrus greening bacterium *Candidatus* Liberibacter asiaticus (Las) exhibits a six-fold starch increase phenotype by using the LasΔ5315 effector to upregulate starch biosynthesis enzymes (ADP-Glucose Pyrophosphorylase (AGPase), GBSS, soluble starch synthase) while downregulating degradation enzymes (α-amylase, α-glucosidase) (Pitino *et al.*, 2018). An early-stage viral infection of several plant RNA and DNA viruses such as cucumber mosaic virus (CMV), tobacco mosaic virus (TMV), potato virus X (PVX), potato virus Y (PVY) and apple geminivirus however shows the opposite pattern of reduced starch accumulation even at low viral titers, with starch biosynthesis genes being transcriptionally downregulated as viruses redirect host carbon for their own replication (Zhao *et al.*, 2022).

Recent computational models predicted that starch metabolism is highly important for how plants grow in response to different nitrogen levels (Tong *et al.*, 2023). However, scientists do not yet fully understand exactly how environmental changes affect starch granule characteristics or whether plants use alternative pathways for starch formation under stress. Some alternative pathways exist, such as transporting different sugar-phosphate molecules across chloroplast membranes (Fettke *et al.*, 2010; Fettke *et al.*, 2011), but their role in metabolic plasticity remains unclear.

Several studies on catalytic and non-catalytic proteins associated with starch metabolism have shown alterations in starch accumulation, starch turnover, starch granule size distribution and granule shape adaptation, as well as granule number and inner starch structure (Fettke & Fernie, 2025). This makes the subject of carbohydrate plasticity fascinating, albeit highly dynamic. Further research is, therefore, needed to understand the molecular mechanisms, genetic basis, and cost–benefit dynamics of carbohydrate plasticity in response to environmental cues.

###### **Photorespiration modulates metabolic plasticity through carbon and nitrogen metabolism**

Photorespiration, also referred to as the C₂ cycle, is a multi-organelle metabolic pathway spanning the chloroplast, peroxisome, and mitochondrion that recycles carbon from 2-phosphoglycolate (2PG), generated by the oxygenation reaction of Rubisco, back into the Calvin cycle (Fernie & Bauwe, 2020; Shi & Bloom, 2021). During this process, 2PG-derived carbon is sequentially converted through glycolate, glyoxylate, glycine, serine, hydroxypyruvate, and glycerate, accompanied by the release and reassimilation of CO₂ and NH₃ before glycerate is returned to the chloroplast as 3-phosphoglycerate, completing the C₂ cycle (Figure 1) (Scherer, 2008; Masclaux-Daubresse *et al.*, 2010; Delin *et al.*, 2012; Zhang *et al.*, 2022). The biochemical reactions, enzymatic components, and subcellular compartmentation of this pathway have been comprehensively characterized (Zayed *et al.*, 2023).

Beyond its core pathway, photorespiration is closely integrated with central plant metabolism through intercompartmental carbon fluxes, glycine–serine interconversion, and ammonium release and reassimilation, thereby linking photosynthetic carbon fixation, nitrogen metabolism, TCA cycle, folate cycle and S-adenosylmethionine (SAM) cycle (Figure 1) (Timm *et al.*, 2008; Bauwe *et al.*, 2010; Liang *et al.*, 2021; Bauwe, 2023; Rosa-Téllez *et al.*, 2024; Sweetlove *et al.*, 2025; Timm *et al.*, 2025). Through this integration, photorespiration functions as a central metabolic hub that coordinates carbon allocation, nitrogen assimilation, and metabolic homeostasis, enabling plants to adjust interconnected metabolic pathways under environmental stress (Timm & Hagemann, 2020; Timm *et al.*, 2021; Bauwe, 2023).

The interaction of photorespiration and carbon metabolism

In addition to its coupling with photosynthesis, photorespiration is tightly integrated with central carbon metabolism, particularly mitochondrial respiration, through coordinated carbon fluxes and redox regulation (Tcherkez *et al.*, 2008). The glycine decarboxylase complex (GDC) forms a key metabolic interface by functionally coupling photorespiration with the tricarboxylic acid (TCA) cycle and mitochondrial electron transport, thereby sustaining NAD⁺ regeneration and photorespiratory carbon flux (Dutilleul *et al.*, 2003; Bykova *et al.*, 2005; Sweetlove *et al.*, 2006). It also provides a major entry point into one-carbon (C1) metabolism through the GDC- and serine hydroxymethyltransferase (SHMT)-mediated conversion of glycine to serine, generating methylene-tetrahydrofolate (CH₂-THF) for folate-mediated one-carbon metabolism and the downstream S-adenosylmethionine (SAM) cycle, thereby linking photorespiratory carbon flow to multiple biosynthetic processes (Figure 1) (Hanson & Roje, 2001; Collakova *et al.*, 2008; Shi & Bloom, 2021). In addition, efficient transport of glycolate and glycerate between the chloroplast, peroxisome, and mitochondrion enables recovery of photorespiratory carbon into the Calvin–Benson cycle, whereas disruption of this transport impairs photorespiratory carbon recycling (Howitz & McCarty, 1985; Pick *et al.*, 2013; Eisenhut *et al.*, 2015).

The interaction of photorespiration and nitrogen metabolism

Photorespiration is tightly coupled to nitrogen metabolism through the substantial release of ammonium during the mitochondrial conversion of glycine to serine by the glycine decarboxylase complex (GDC) and serine hydroxymethyltransferase (SHMT) (Figure 1) (Keys *et al.*, 1978; Lea *et al.*, 1992; Wingler *et al.*, 2000; Bauwe & Kolukisaoglu, 2003). This photorespiratory ammonium flux can exceed primary nitrate assimilation by up to an order of magnitude, making rapid reassimilation via the glutamine synthetase–glutamate synthase (GS/GOGAT) cycle essential for nitrogen recycling and amino acid metabolism (Bauwe *et al.*, 2010; Eisenhut *et al.*, 2015; Aroca *et al.*, 2023). Photorespiration is also closely associated with nitrate assimilation in C₃ plants, and conditions that suppress photorespiration, such as elevated CO₂ or reduced O₂, inhibit nitrate reduction and assimilation, partly because photorespiration contributes to the cytosolic NADH supply for nitrate and nitrite reduction (Bloom *et al.*, 1989; Bloom *et al.*, 2002; Rachmilevitch *et al.*, 2004; Bloom *et al.*, 2010; Carlisle *et al.*, 2014; Bloom, 2015; Aroca *et al.*, 2023). Consequently, elevated CO₂ is frequently associated with reduced plant nitrogen content and increased C/N ratios, although the underlying mechanisms remain under debate(Aroca *et al.*, 2023). Furthermore, the GS/GOGAT cycle further links photorespiratory nitrogen metabolism with central carbon metabolism through its requirement for 2-oxoglutarate as the carbon skeleton (Figure 1), and perturbation of photorespiratory ammonium reassimilation is accompanied by extensive metabolic and transcriptional reprogramming across both carbon and nitrogen metabolism (Bauwe *et al.*, 2010; Aroca *et al.*, 2023).

Photorespiration as a Driver of Metabolic Plasticity under Abiotic Stress

Under abiotic stress, plants adjust photorespiratory flux in response to stress-induced changes in internal gas exchange, which suppress photosynthetic carbon assimilation while promoting RuBP oxygenation. Enhanced turnover of 2-phosphoglycolate (2PG) prevents inhibition of carbon fixation and maintains carbon flux through central metabolism under high light, elevated temperature, and drought conditions (Voss *et al.*, 2013; Chastain *et al.*, 2014; Flugel *et al.*, 2017; Levey *et al.*, 2019). As a key photorespiratory intermediate, 2PG regulates multiple carbon metabolic pathways by inhibiting triosephosphate isomerase, sedoheptulose-1,7-bisphosphatase (SBPase), and phosphofructokinase, thereby influencing Calvin–Benson cycle activity, starch and sucrose metabolism, and glycolysis (Chollet & Anderson, 1976; Kelly & Latzko, 1976; Flugel *et al.*, 2017; Li *et al.*, 2019). In parallel, photorespiration regulates cellular redox and energy metabolism through mitochondrial glycine-to-serine conversion and NADH production, supporting mitochondrial electron transport and ATP synthesis under stress (Figure 1) (Bourguignon *et al.*, 1988; Hasse *et al.*, 2013; Reinholdt *et al.*, 2019; da Fonseca-Pereira *et al.*, 2020). Consistently, enhanced activities of phosphoglycolate phosphatase (PGLP) and glycolate oxidase (GOX) improve metabolic performance and abiotic stress tolerance when photorespiratory flux is elevated (Cui *et al.*, 2016; Flugel *et al.*, 2017; Timm *et al.*, 2019).

Stress-induced modulation of photorespiration also propagates into nitrogen metabolism through changes in ammonium release, amino acid interconversion, and one-carbon metabolism. The mitochondrial conversion of glycine to serine represents a major source of ammonium and one-carbon units, directly coupling photorespiratory flux with nitrogen reassimilation and C/N balance (Timm *et al.*, 2021). Consequently, changes in photorespiratory flux alter the turnover of glycine, serine, glutamine, and glutamate, influencing carbon skeleton availability, nitrogen assimilation, and cellular redox status (Timm & Hagemann, 2020). Enhanced photorespiration further promotes ammonium reassimilation through the GS/GOGAT cycle, alleviating C/N imbalance when nitrogen assimilation becomes limiting under abiotic stress (Figure 1) (Hodges *et al.*, 2016). These adjustments integrate photorespiration with photosynthesis, respiration, and nitrogen metabolism during prolonged or combined stresses (Dutilleul *et al.*, 2005; Hager *et al.*, 2010; Rosa-Téllez *et al.*, 2024).

**Figure 1: Proposed model for the connection between the photorespiratory pathway and other primary metabolic pathways. Photorespiration is initiated by the production of the toxic compound 2PG from RuBP and O₂, catalyzed by Rubisco. Through a series of reactions, the pathway proceeds via the peroxisome and the glycine-to-serine conversion system in the mitochondrion, before returning to the chloroplast via the peroxisome and being converted to 3PGA to sustain the Calvin–Benson cycle. Enzymes: AAT, alanine aminotransferase; CAT, catalase; GDC, glycine decarboxylase complex; GGAT (GGT), glutamate-glyoxylate aminotransferase; GLYK, glycerate kinase; GOGAT, glutamine oxoglutarate aminotransferase; GOX, glycolate oxidase; GS2, glutamine synthetase 2; HPR1, hydroxypyruvate reductase 1; MTHFR, methylenetetrahydrofolate reductase; NR, nitrate reductase; NiR, nitrite reductase; PGLP, 2-phosphoglycolate phosphatase; Rubisco, ribulose-1,5-bisphosphate carboxylase/oxygenase; SERAT, serine acetyltransferase; SGT (SGAT), serine:glyoxylate aminotransferase; SHMT1, serine hydroxymethyltransferase 1 (mitochondrial); SHMT5, serine hydroxymethyltransferase 5 (chloroplastic). Metabolites: 2-OG, 2-oxoglutarate; 2PG, 2-phosphoglycolate; 3PGA, 3-phosphoglycerate; ADP, adenosine diphosphate; Ala, alanine; Asp, aspartate; ATP, adenosine triphosphate; CH₂-THF, 5,10-methylenetetrahydrofolate; CH₃-THF, 5-methyltetrahydrofolate; Cys, cysteine; Fru6P, fructose-6-phosphate; Gln, glutamine; Glu, glutamate; Gly, glycine; Glycolate; GTP, guanosine triphosphate; Methionine; NAD⁺, nicotinamide adenine dinucleotide (oxidized); NADH, nicotinamide adenine dinucleotide (reduced); NH₄⁺, ammonium; OAS, O-acetylserine; PEP, phosphoenolpyruvate; Pi, inorganic phosphate; Pyr, pyruvate; RuBP, ribulose-1,5-bisphosphate; SAH, S-adenosylhomocysteine; SAM, S-adenosylmethionine; Ser, serine; THF, tetrahydrofolate; Triose, triose phosphate.**

***Lipid-derived hormone genes regulate lipid metabolic plasticity***

Beyond structural remodeling of membrane and storage lipids, lipid-derived hormones and oxylipins provide an additional regulatory layer linking lipid remodeling to stress adaptation. Under temperature stress, abscisic acid (ABA) and jasmonates (JAs) are particularly well characterized. In rice, the ABA biosynthetic genes OsNCED1 and OsNCED4 enhance cold tolerance, at least in part through modulation of reactive oxygen species homeostasis (Xiang *et al.*, 2024; Xiang *et al.*, 2026). In parallel, JA biosynthesis under cold stress promotes expression of the long-chain base Δ8 desaturase **SLD1** in Arabidopsis thaliana, thereby enhancing sphingolipid desaturation and cold resilience (Huang *et al.*, 2025). Under heat stress, signaling promotes heat resistance through the heat-shock pathway involving HSFA2, whereas ABA signaling promotes heat resistance through regulators including HSFA6A; in addition, overexpression of the BR biosynthetic gene DWF4 enhances seedling thermotolerance in *Brassica napus* (Clarke *et al.*, 2009; Huang *et al.*, 2016; Sahni *et al.*, 2016). Lipid-derived oxylipins also play important roles in biotic stress-associated plasticity. In Arabidopsis, esterified oxylipins such as **Arabidopsides**, including OPDA- and dnOPDA-containing MGDG and DGDG species, accumulate during hypersensitive responses and pathogen infection. Their dynamics during Botrytis cinerea infection suggest that galactolipid-bound oxylipins may serve not only as precursors for JA production but also as defence-active lipid species in their own right (Kourtchenko *et al.*, 2007).

Collectively, these findings place lipid-derived hormones and oxylipins at the interface between lipid remodeling and stress signaling. By integrating changes in lipid precursor availability with hormonal and defence pathways, they expand the regulatory scope of lipid metabolic plasticity beyond membrane structure and storage dynamics alone.

**Literature Cited**

**Abt, M.R., Pfister, B., Sharma, M., Eicke, S., Bürgy, L., Neale, I., Seung, D. & Zeeman, S.C.** (2020) STARCH SYNTHASE5, a Noncanonical Starch Synthase-Like Protein, Promotes Starch Granule Initiation in Arabidopsis. *The Plant Cell*, **32**, 2543–2565.

**Abt, M.R. & Zeeman, S.C.** (2020) Evolutionary innovations in starch metabolism. *Current Opinion in Plant Biology*, **55**, 109–117.

**Adams III, W.W., Stewart, J.J., Cohu, C.M., Muller, O. & Demmig-Adams, B.** (2016) Habitat temperature and precipitation of Arabidopsis thaliana ecotypes determine the response of foliar vasculature, photosynthesis, and transpiration to growth temperature. *Frontiers in Plant Science*, **7**, 1026.

**Apriyanto, A., Compart, J. & Fettke, J.** (2022) A review of starch, a unique biopolymer – Structure, metabolism and in planta modifications. *Plant Science*, **318**, 111223.

**Aroca, A., García-Díaz, I., García-Calderón, M., Gotor, C., Márquez, A. & Betti, M.** (2023) Photorespiration: regulation and new insights on the potential role of persulfidation. *Journal of Experimental Botany*, **74**, 6023–6039.

**Bauwe, H.** (2023) Photorespiration-Rubisco's repair crew. *Journal of Plant Physiology*, **280**, 31.

**Bauwe, H., Hagemann, M. & Fernie, A.** (2010) Photorespiration: players, partners and origin. *Trends in Plant Science*, **15**, 330–336.

**Bauwe, H. & Kolukisaoglu, Ü.** (2003) Genetic manipulation of glycine decarboxylation. *Journal of Experimental Botany*, **54**, 1523–1535.

**Bloom, A.J.** (2015) Photorespiration and nitrate assimilation: a major intersection between plant carbon and nitrogen. *Photosynthesis research*, **123**, 117–128.

**Bloom, A.J., Burger, M., Asensio, J.S.R. & Cousins, A.B.** (2010) Carbon dioxide enrichment inhibits nitrate assimilation in wheat and Arabidopsis. *Science*, **328**, 899–903.

**Bloom, A.J., Caldwell, R.M., Finazzo, J., Warner, R.L. & Weissbart, J.** (1989) Oxygen and carbon dioxide fluxes from barley shoots depend on nitrate assimilation. *Plant Physiology*, **91**, 352–356.

**Bloom, A.J., Smart, D.R., Nguyen, D.T. & Searles, P.S.** (2002) Nitrogen assimilation and growth of wheat under elevated carbon dioxide. *Proceedings of the National Academy of Sciences*, **99**, 1730–1735.

**Bourguignon, J., Neuburger, M. & Douce, R.** (1988) Resolution and Characterization of the Glycine-Cleavage Reaction in Pea Leaf Mitochondria - Properties of the Forward Reaction Catalyzed by Glycine Decarboxylase and Serine Hydroxymethyltransferase. *Biochem J*, **255**, 169–178.

**Bykova, N.V., Keerberg, O., Pärnik, T., Bauwe, H. & Gardeström, P.** (2005) Interaction between photorespiration and respiration in transgenic potato plants with antisense reduction in glycine decarboxylase. *Planta*, **222**, 130–140.

**Carlisle, E., Yarnes, C., Toney, M.D. & Bloom, A.J.** (2014) Nitrate reductase 15N discrimination in Arabidopsis thaliana, Zea mays, Aspergillus niger, Pichea angusta, and Escherichia coli. *Frontiers in plant science*, **5**, 317.

**Chastain, D.R., Snider, J.L., Collins, G.D., Perry, C.D., Whitaker, J. & Byrd, S.A.** (2014) Water deficit in field-grown Gossypium hirsutum primarily limits net photosynthesis by decreasing stomatal conductance, increasing photorespiration, and increasing the ratio of dark respiration to gross photosynthesis. *Journal of Plant Physiology*, **171**, 1576–1585.

**Chollet, R. & Anderson, L.L.** (1976) Regulation of ribulose 1,5-bisphosphate carboxylase-oxygenase activities by temperature pretreatment and chloroplast metabolites. *Archives of Biochemistry and Biophysics*, **176**, 344–351.

**Clarke, S.M., Cristescu, S.M., Miersch, O., Harren, F.J.M., Wasternack, C. & Mur, L.A.J.** (2009) Jasmonates act with salicylic acid to confer basal thermotolerance in Arabidopsis thaliana. *New Phytologist*, **182**, 175–187.

**Collakova, E., Goyer, A., Naponelli, V., Krassovskaya, I., Gregory III, J.F., Hanson, A.D. & Shachar-Hill, Y.** (2008) Arabidopsis 10-formyl tetrahydrofolate deformylases are essential for photorespiration. *The Plant Cell*, **20**, 1818–1832.

**Compart, J., Singh, A., Fettke, J. & Apriyanto, A.** (2023) Customizing Starch Properties: A Review of Starch Modifications and Their Applications. *Polymers*, **15**, 3491.

**CUELLAR‐ORTIZ, S.M., DE LA PAZ ARRIETA‐MONTIEL, M., ACOSTA‐GALLEGOS, J. & Covarrubias, A.A.** (2008) Relationship between carbohydrate partitioning and drought resistance in common bean. *Plant, cell & environment*, **31**, 1399–1409.

**Cui, L.L., Lu, Y.S., Li, Y., Yang, C. & Peng, X.X.** (2016) Overexpression of Glycolate Oxidase Confers Improved Photosynthesis under High Light and High Temperature in Rice. *Frontiers in Plant Science*, **7**, 1165.

**da Fonseca-Pereira, P., Souza, P.V.L., Hou, L.Y., Schwab, S., Geigenberger, P., Nunes-Nesi, A., Timm, S., Fernie, A.R., Thormahlen, I., Araujo, W.L. & Daloso, D.M.** (2020) Thioredoxin h2 contributes to the redox regulation of mitochondrial photorespiratory metabolism. *Plant, Cell & Environment*, **43**, 188–208.

**David, L.C., Lee, S.K., Bruderer, E., Abt, M.R., Fischer-Stettler, M., Tschopp, M.A., Solhaug, E.M., Sanchez, K. & Zeeman, S.C.** (2022) BETA-AMYLASE9 is a plastidial nonenzymatic regulator of leaf starch degradation. *Plant Physiol*, **188**, 191–207.

**Delin, S., Stenberg, B., Nyberg, A. & Brohede, L.** (2012) Potential methods for estimating nitrogen fertilizer value of organic residues. *Soil Use and Management*, **28**, 283–291.

**Dutilleul, C., Driscoll, S., Cornic, G., De Paepe, R., Foyer, C.H. & Noctor, G.** (2003) Functional mitochondrial complex I is required by tobacco leaves for optimal photosynthetic performance in photorespiratory conditions and during transients. *Plant Physiology*, **131**, 264–275.

**Dutilleul, C., Lelarge, C., Prioul, J.L., De Paepe, R., Foyer, C.H. & Noctor, G.** (2005) Mitochondria-driven changes in leaf NAD status exert a crucial influence on the control of nitrate assimilation and the integration of carbon and nitrogen metabolism. *Plant Physiology*, **139**, 64–78.

**Eisenhut, M., Hocken, N. & Weber, A.** (2015) Plastidial metabolite transporters integrate photorespiration with carbon, nitrogen, and sulfur metabolism. *Cell Calcium*, **58**, 98–104.

**Ericsson, T.** (1995) Growth and shoot: root ratio of seedlings in relation to nutrient availability. *Plant and Soil*, **168**, 205–214.

**Fernie, A.R. & Bauwe, H.** (2020) Wasteful, essential, evolutionary stepping stone? The multiple personalities of the photorespiratory pathway. *The Plant Journal*, **102**, 666–677.

**Fernie, A.R. & Tohge, T.** (2013) Plastic, fantastic! Phenotypic variance in the transcriptional landscape of the grape berry. *Genome Biology*, **14**, 119.

**Fernie, A.R. & Tohge, T.** (2017) The Genetics of Plant Metabolism. *Annual Review of Genetics*, **51**, 287–310.

**Fettke, J., Albrecht, T., Hejazi, M., Mahlow, S., Nakamura, Y. & Steup, M.** (2010) Glucose 1-phosphate is efficiently taken up by potato (Solanum tuberosum) tuber parenchyma cells and converted to reserve starch granules. *New Phytologist*, **185**, 663–675.

**Fettke, J., Eckermann, N., Tiessen, A., Geigenberger, P. & Steup, M.** (2005) Identification, subcellular localization and biochemical characterization of water-soluble heteroglycans (SHG) in leaves of Arabidopsis thaliana L.: distinct SHG reside in the cytosol and in the apoplast. *The Plant Journal*, **43**, 568–585.

**Fettke, J. & Fernie, A.R.** (2025) Do storage reserves contribute to plant phenotypic plasticity? *Trends in Plant Science*, **30**, 364–372.

**Fettke, J., Malinova, I., Albrecht, T., Hejazi, M. & Steup, M.** (2011) Glucose-1-phosphate transport into protoplasts and chloroplasts from leaves of Arabidopsis. *Plant Physiol*, **155**, 1723–1734.

**Flugel, F., Timm, S., Arrivault, S., Florian, A., Stitt, M., Fernie, A.R. & Bauwe, H.** (2017) The Photorespiratory Metabolite 2-Phosphoglycolate Regulates Photosynthesis and Starch Accumulation in Arabidopsis. *The Plant Cell*, **29**, 2537–2551.

**González-Cruz, J. & Pastenes, C.** (2012) Water-stress-induced thermotolerance of photosynthesis in bean (Phaseolus vulgaris L.) plants: The possible involvement of lipid composition and xanthophyll cycle pigments. *Environmental and Experimental Botany*, **77**, 127–140.

**Hager, J., Pellny, T.K., Mauve, C., Lelarge-Trouverie, C., De Paepe, R., Foyer, C.H. & Noctor, G.** (2010) Conditional modulation of NAD levels and metabolite profiles in Nicotiana sylvestris by mitochondrial electron transport and carbon/nitrogen supply. *Planta*, **231**, 1145–1157.

**Hanson, A.D. & Roje, S.** (2001) One-carbon metabolism in higher plants. *Annual review of plant biology*, **52**, 119–137.

**Hasse, D., Andersson, E., Carlsson, G., Masloboy, A., Hagemann, M., Bauwe, H. & Andersson, I.** (2013) Structure of the homodimeric glycine decarboxylase P-protein from Synechocystis sp. PCC 6803 suggests a mechanism for redox regulation. *Journal of Biological Chemistry*, **288**, 35333–35345.

**Herman, D.J., Knowles, L.O. & Knowles, N.R.** (2017) Heat stress affects carbohydrate metabolism during cold-induced sweetening of potato (Solanum tuberosum L.). *Planta*, **245**, 563–582.

**Hodges, M., Dellero, Y., Keech, O., Betti, M., Raghavendra, A., Sage, R., Zhu, X., Allen, D. & Weber, A.** (2016) Perspectives for a better understanding of the metabolic integration of photorespiration within a complex plant primary metabolism network. *Journal of Experimental Botany*, **67**, 3015–3026.

**Howitz, K.T. & McCarty, R.E.** (1985) Substrate specificity of the pea chloroplast glycolate transporter. *Biochemistry*, **24**, 3645–3650.

**Huang, L.Q., Yang, C., Iqbal, A., Liu, J.T., Li, Y.K., Bao, H.N., Liu, H.J., Chen, Y.L., Li, J., Zhang, K., Li, S., Zhao, X., Hao, X., Tian, L., Li, D. & Yao, N.** (2025) Jasmonate pathway regulates sphingolipid desaturation during cold stress. *New Phytologist*, **247**, 191–208.

**Huang, Y.C., Niu, C.Y., Yang, C.R. & Jinn, T.L.** (2016) The Heat Stress Factor HSFA6b Connects ABA Signaling and ABA-Mediated Heat Responses. *Plant Physiology*, **172**, 1182–1199.

**Kamble, N.U., Makhamadjonov, F., Fahy, B., Martins, C., Saalbach, G. & Seung, D.** (2023) Initiation of B-type starch granules in wheat endosperm requires the plastidial α-glucan phosphorylase PHS1. *The Plant Cell*, **35**, 4091–4110.

**Kaplan, F., Kopka, J., Sung, D.Y., Zhao, W., Popp, M., Porat, R. & Guy, C.L.** (2007) Transcript and metabolite profiling during cold acclimation of Arabidopsis reveals an intricate relationship of cold‐regulated gene expression with modifications in metabolite content. *The Plant Journal*, **50**, 967–981.

**Kelly, G.J. & Latzko, E.** (1976) Inhibition of spinach-leaf phosphofructokinase by 2-phosphoglycollate. *FEBS Lett*, **68**, 55–58.

**Keys, A., Bird, I., Cornelius, M., Lea, P., Wallsgrove, R.M. & Miflin, B.** (1978) Photorespiratory nitrogen cycle. *Nature*, **275**, 741–743.

**Koper, K., Hwang, S.-K., Wood, M., Singh, S., Cousins, A., Kirchhoff, H. & Okita, T.W.** (2020) The Rice Plastidial Phosphorylase Participates Directly in Both Sink and Source Processes. *Plant and Cell Physiology*, **62**, 125–142.

**Kourtchenko, O., Andersson, M.X., Hamberg, M., Brunnstrom, A., Gobel, C., McPhail, K.L., Gerwick, W.H., Feussner, I. & Ellerstrom, M.** (2007) Oxo-phytodienoic acid-containing galactolipids in Arabidopsis: jasmonate signaling dependence. *Plant Physiology*, **145**, 1658–1669.

**Krasensky, J. & Jonak, C.** (2012) Drought, salt, and temperature stress-induced metabolic rearrangements and regulatory networks. *Journal of experimental botany*, **63**, 1593–1608.

**Lea, P.J., Blackwell, R.D. & Joy, K.W.** (1992) Ammonia Assimilation in Higher-Plants. *Pr Phyt Soc*, **33**, 153–186.

**Lee, Y.J., Zhang, D., Stolze, S.C., Saridis, G., Ebert, M.K., Nakagami, H. & Doehlemann, G.** (2026) Ustilago maydis disrupts carbohydrate signaling networks to induce hypertrophy in host cells. *Nature Communications*.

**Levey, M., Timm, S., Mettler-Altmann, T., Luca Borghi, G., Koczor, M., Arrivault, S., Pm Weber, A., Bauwe, H., Gowik, U. & Westhoff, P.** (2019) Efficient 2-phosphoglycolate degradation is required to maintain carbon assimilation and allocation in the C4 plant Flaveria bidentis. *Journal of Biological Chemistry*, **70**, 575–587.

**Li, J., Weraduwage, S.M., Preiser, A.L., Tietz, S., Weise, S.E., Strand, D.D., Froehlich, J.E., Kramer, D.M., Hu, J. & Sharkey, T.D.** (2019) A Cytosolic Bypass and G6P Shunt in Plants Lacking Peroxisomal Hydroxypyruvate Reductase. *Plant Physiology*, **180**, 783–792.

**Li, X., Apriyanto, A., Castellanos, J.F., Compart, J., Muntaha, S.N. & Fettke, J.** (2022) Dpe2/phs1 revealed unique starch metabolism with three distinct phases characterized by different starch granule numbers per chloroplast, allowing insights into the control mechanism of granule number regulation by gene co-regulation and metabolic profiling. *Frontiers in Plant Science*, **13**, 1039534.

**Liang, Y., Wang, J., Zeng, F., Wang, Q., Zhu, L., Li, H., Guo, N. & Chen, H.** (2021) Photorespiration Regulates Carbon-Nitrogen Metabolism by Magnesium Chelatase D Subunit in Rice. *J. Agric. Food Chem.*, **69**, 112–125.

**Lin, Q., Xie, Y., Guan, W., Duan, Y., Wang, Z. & Sun, C.** (2019) Combined transcriptomic and proteomic analysis of cold stress induced sugar accumulation and heat shock proteins expression during postharvest potato tuber storage. *Food Chemistry*, **297**, 124991.

**Liu, C., Pfister, B., Osman, R., Ritter, M., Heutinck, A., Sharma, M., Eicke, S., Fischer-Stettler, M., Seung, D., Bompard, C., Abt, M.R. & Zeeman, S.C.** (2023) LIKE EARLY STARVATION 1 and EARLY STARVATION 1 promote and stabilize amylopectin phase transition in starch biosynthesis. *Science Advances*, **9**, eadg7448.

**Liu, Q., Li, X. & Fettke, J.** (2021) Starch Granules in Arabidopsis thaliana Mesophyll and Guard Cells Show Similar Morphology but Differences in Size and Number. *International Journal of Molecular Sciences*, **22**, 5666.

**Mahlow, S., Hejazi, M., Kuhnert, F., Garz, A., Brust, H., Baumann, O. & Fettke, J.** (2014) Phosphorylation of transitory starch by α-glucan, water dikinase during starch turnover affects the surface properties and morphology of starch granules. *New Phytologist*, **203**, 495–507.

**Malinova, I., Alseekh, S., Feil, R., Fernie, A.R., Baumann, O., Schöttler, M.A., Lunn, J.E. & Fettke, J.** (2017) Starch Synthase 4 and Plastidal Phosphorylase Differentially Affect Starch Granule Number and Morphology. *Plant Physiology*, **174**, 73–85.

**Malinova, I., Qasim, H.M., Brust, H. & Fettke, J.** (2018) Parameters of Starch Granule Genesis in Chloroplasts of Arabidopsis thaliana. *Frontiers in Plant Science*, **9**, 761.

**Masclaux-Daubresse, C., Daniel-Vedele, F., Dechorgnat, J., Chardon, F., Gaufichon, L. & Suzuki, A.** (2010) Nitrogen uptake, assimilation and remobilization in plants: challenges for sustainable and productive agriculture. *Annals of botany*, **105**, 1141–1157.

**Mérida, A. & Fettke, J.** (2021) Starch granule initiation in Arabidopsis thaliana chloroplasts. *The Plant Journal*, **107**, 688–697.

**Pick, T.R., Bräutigam, A., Schulz, M.A., Obata, T., Fernie, A.R. & Weber, A.P.** (2013) PLGG1, a plastidic glycolate glycerate transporter, is required for photorespiration and defines a unique class of metabolite transporters. *Proceedings of the National Academy of Sciences*, **110**, 3185–3190.

**Pitino, M., Allen, V. & Duan, Y.** (2018) LasΔ5315 effector induces extreme starch accumulation and chlorosis as Ca. Liberibacter asiaticus infection in Nicotiana benthamiana. *Frontiers in Plant Science*, **9**, 113.

**Poorter, H. & Nagel, O.** (2000) The role of biomass allocation in the growth response of plants to different levels of light, CO2, nutrients and water: a quantitative review. *Australian Journal of Plant Physiology*, **27**, 1191–1191.

**Rachmilevitch, S., Cousins, A.B. & Bloom, A.J.** (2004) Nitrate assimilation in plant shoots depends on photorespiration. *Proceedings of the National Academy of Sciences*, **101**, 11506–11510.

**Reinholdt, O., Schwab, S., Zhang, Y., Reichheld, J.P., Fernie, A.R., Hagemann, M. & Timm, S.** (2019) Redox-Regulation of Photorespiration through Mitochondrial Thioredoxin o1. *Plant Physiology*, **181**, 442–457.

**Ribeiro, C., Stitt, M. & Hotta, C.T.** (2022) How stress affects your budget—stress impacts on starch metabolism. *Frontiers in plant science*, **13**, 774060.

**Roldán, I., Wattebled, F., Mercedes Lucas, M., Delvallé, D., Planchot, V., Jiménez, S., Pérez, R., Ball, S., D'Hulst, C. & Mérida, A.** (2007) The phenotype of soluble starch synthase IV defective mutants of Arabidopsis thaliana suggests a novel function of elongation enzymes in the control of starch granule formation. *The Plant Journal*, **49**, 492–504.

**Rosa-Téllez, S., Alcántara-Enguídanos, A., Martínez-Seidel, F., Casatejada-Anchel, R., Saeheng, S., Bailes, C.L., Erban, A., Barbosa-Medeiros, D., Alepúz, P., Matus, J.T., Kopka, J., Muñoz-Bertomeu, J., Krueger, S., Roje, S., Fernie, A.R. & Ros, R.** (2024) The serine-glycine-one-carbon metabolic network orchestrates changes in nitrogen and sulfur metabolism and shapes plant development. *The Plant Cell*, **36**, 404–426.

**Sahni, S., Prasad, B.D., Liu, Q., Grbic, V., Sharpe, A., Singh, S.P. & Krishna, P.** (2016) Overexpression of the brassinosteroid biosynthetic gene DWF4 in Brassica napus simultaneously increases seed yield and stress tolerance. *Scientific Reports*, **6**, 28298.

**Scherer, H.W.** (2008) Impact of sulfur on N2 fixation of legumes. In *Sulfur assimilation and abiotic stress in plants*: Springer, pp. 43–54.

**Seung, D., Orzechowski, S. & Fettke, J.** (2025) Old player, new roles: defining the role of the plastidial phosphorylase. *New Phytologist*, **247**, 1622–1632.

**Seung, D. & Smith, A.M.** (2019) Starch granule initiation and morphogenesis-progress in Arabidopsis and cereals. *Journal of Evolutionary Biology*, **70**, 771–784.

**Sharma, M., Abt, M.R., Eicke, S., Ilse, T.E., Liu, C., Lucas, M.S., Pfister, B. & Zeeman, S.C.** (2024) MFP1 defines the subchloroplast location of starch granule initiation. *Proceedings of the National Academy of Sciences*, **121**, e2309666121.

**Sharma, S., Friberg, M., Vogel, P., Turesson, H., Olsson, N., Andersson, M. & Hofvander, P.** (2023) Pho1a (plastid starch phosphorylase) is duplicated and essential for normal starch granule phenotype in tubers of Solanum tuberosum L. *Frontiers in Plant Science*, **14**, 1220973.

**Shi, X. & Bloom, A.** (2021) Photorespiration: The Futile Cycle? *Plants*, **10**, 18.

**Singh, A., Compart, J., Al-Rawi, S.A., Mahto, H., Ahmad, A.M. & Fettke, J.** (2022) LIKE EARLY STARVATION 1 alters the glucan structures at the starch granule surface and thereby influences the action of both starch-synthesizing and starch-degrading enzymes. *The Plant Journal*, **111**, 819–835.

**Sweetlove, L.J., Lytovchenko, A., Morgan, M., Nunes-Nesi, A., Taylor, N.L., Baxter, C.J., Eickmeier, I. & Fernie, A.R.** (2006) Mitochondrial uncoupling protein is required for efficient photosynthesis. *Proceedings of the National Academy of Sciences*, **103**, 19587–19592.

**Sweetlove, L.J., Ratcliffe, R.G. & Fernie, A.R.** (2025) Non-canonical plant metabolism. *Nature Plants*, **11**, 696–708.

**Tcherkez, G., Bligny, R., Gout, E., Mahé, A., Hodges, M. & Cornic, G.** (2008) Respiratory metabolism of illuminated leaves depends on CO<sub>2</sub> and O<sub>2</sub> conditions. *Proceedings of the National Academy of Sciences*, **105**, 797–802.

**Thalmann, M. & Santelia, D.** (2017) Starch as a determinant of plant fitness under abiotic stress. *New phytologist*, **214**, 943–951.

**Timm, S. & Hagemann, M.** (2020) Photorespiration-how is it regulated and how does it regulate overall plant metabolism? *Journal of Experimental Botany*, **71**, 3955–3965.

**Timm, S., Nunes-Nesi, A., Florian, A., Eisenhut, M., Morgenthal, K., Wirtz, M., Hell, R., Weckwerth, W., Hagemann, M., Fernie, A. & Bauwe, H.** (2021) Metabolite Profiling in Arabidopsis thaliana with Moderately Impaired Photorespiration Reveals Novel Metabolic Links and Compensatory Mechanisms of Photorespiration. *Metabolites*, **11**, 21.

**Timm, S., Nunes-Nesi, A., Parnik, T., Morgenthal, K., Wienkoop, S., Keerberg, O., Weckwerth, W., Kleczkowski, L.A., Fernie, A.R. & Bauwe, H.** (2008) A cytosolic pathway for the conversion of hydroxypyruvate to glycerate during photorespiration in Arabidopsis. *The Plant Cell*, **20**, 2848–2859.

**Timm, S., Sun, H., Hagemann, M., Huang, W. & Fernie, A.R.** (2025) An old dog with new tricks-the value of photorespiration as a central metabolic hub with implications for environmental acclimation. *Plant Physiol*, **198**.

**Timm, S., Woitschach, F., Heise, C., Hagemann, M. & Bauwe, H.** (2019) Faster Removal of 2-Phosphoglycolate through Photorespiration Improves Abiotic Stress Tolerance of Arabidopsis. *Plants*, **8**, 563.

**Tong, H., Laitinen, R.A.E. & Nikoloski, Z.** (2023) Predicting plasticity of rosette growth and metabolic fluxes in Arabidopsis thaliana. *New Phytol*, **240**, 426–438.

**Voss, I., Sunil, B., Scheibe, R. & Raghavendra, A.S.** (2013) Emerging concept for the role of photorespiration as an important part of abiotic stress response. *Plant Biol.*, **15**, 713–722.

**Wingler, A., Lea, P.J., Quick, W.P. & Leegood, R.C.** (2000) Photorespiration: metabolic pathways and their role in stress protection. *Philosophical Transactions of the Royal Society of London. Series B: Biological Sciences*, **355**, 1517–1529.

**Xiang, Z., Lu, M., Yao, Y., Zhang, M., Zhang, Z., Chen, S., Pan, X., Fu, H. & Zhang, L.** (2026) OsNCED1, a chloroplast ABA biosynthase, regulates multiple abiotic stress tolerance in rice. *Plant Science*, **364**, 112979.

**Xiang, Z., Zhang, L., Long, Y., Zhang, M., Yao, Y., Deng, H., Quan, C., Lu, M., Cui, B. & Wang, D.** (2024) An ABA biosynthesis enzyme gene OsNCED4 regulates NaCl and cold stress tolerance in rice. *Scientific reports*, **14**, 26711.

**Xu, X., Dees, D., Dechesne, A., Huang, X.-F., Visser, R.G.F. & Trindade, L.M.** (2017) Starch phosphorylation plays an important role in starch biosynthesis. *Carbohydrate Polymers*, **157**, 1628–1637.

**You, Y., Zhang, M., Yang, W., Li, C., Liu, Y., Li, C., He, J. & Wu, W.** (2020) Starch phosphorylation and the in vivo regulation of starch metabolism and characteristics. *International Journal of Biological Macromolecules*, **159**, 823–831.

**Zayed, O., Hewedy, O.A., Abdelmoteleb, A., Ali, M., Youssef, M.S., Roumia, A.F., Seymour, D. & Yuan, Z.-C.** (2023) Nitrogen Journey in Plants: From Uptake to Metabolism, Stress Response, and Microbe Interaction. *Biomolecules*, **13**, 1443.

**Zhang, S., Liu, Y., Du, M., Shou, G., Wang, Z. & Xu, G.** (2022) Nitrogen as a regulator for flowering time in plant. *Plant and Soil*, **480**, 1–29.

**Zhao, W., Wang, L., Liu, M., Zhang, D., Andika, I.B., Zhu, Y. & Sun, L.** (2022) A reduced starch level in plants at early stages of infection by viruses can be considered a broad-range indicator of virus presence. *Viruses*, **14**, 1176.
